# Mesenchymal Stem Cell Membrane‐Camouflaged Liposomes for Biomimetic Delivery of Cyclosporine A for Hepatic Ischemia‐Reperfusion Injury Prevention

**DOI:** 10.1002/advs.202404171

**Published:** 2024-06-20

**Authors:** Haitian Chen, Wen Yin, Kang Yao, Jinliang Liang, Jianye Cai, Xin Sui, Xuegang Zhao, Jiebin Zhang, Jiaqi Xiao, Rong Li, Qiuli Liu, Jia Yao, Guohua You, Yasong Liu, Chenhao Jiang, Xiaotong Qiu, Tingting Wang, Qiang You, Yingcai Zhang, Mo Yang, Jun Zheng, Zong Dai, Yang Yang

**Affiliations:** ^1^ Department of Hepatic Surgery and Liver Transplantation Center of The Third Affiliated Hospital Organ Transplantation Institute Sun Yat‐sen University Organ Transplantation Research Center of Guangdong Province Guangdong Province Engineering Laboratory for Transplantation Medicine Guangzhou 510630 China; ^2^ Guangdong Key Laboratory of Liver Disease Research The Third Affiliated Hospital of Sun Yat‐sen University Guangzhou 510630 China; ^3^ School of Biomedical Engineering Shenzhen Campus of Sun Yat‐sen University Shenzhen 518107 China; ^4^ Department of Biomedical Engineering The Hong Kong Polytechnic University Hong Kong 999077 China; ^5^ Guangdong province engineering laboratory for transplantation medicine Guangzhou China; ^6^ Surgical ICU The Third Affiliated Hospital of Sun Yat‐sen University Guangzhou 510630 China; ^7^ The Biotherapy Center the Third Affiliated Hospital of Sun Yat‐Sen University Guangzhou 510630 China; ^8^ Department of Hepatobiliary Surgery People's Hospital of Xinjiang Uyghur Autonomous Region Urumqi 830001 China

**Keywords:** biomimetic delivery, cyclosporine A, ischemia‐reperfusion injury, mesenchymal stem cell

## Abstract

Hepatic ischemia‐reperfusion injury (HIRI) is a prevalent issue during liver resection and transplantation, with currently no cure or FDA‐approved therapy. A promising drug, Cyclosporin A (CsA), ameliorates HIRI by maintaining mitochondrial homeostasis but has systemic side effects due to its low bioavailability and high dosage requirements. This study introduces a biomimetic CsA delivery system that directly targets hepatic lesions using mesenchymal stem cell (MSC) membrane‐camouflaged liposomes. These hybrid nanovesicles (NVs), leveraging MSC‐derived proteins, demonstrate efficient inflammatory chemotaxis, transendothelial migration, and drug‐loading capacity. In a HIRI mouse model, the biomimetic NVs accumulated at liver injury sites entered hepatocytes, and significantly reduced liver damage and restore function using only one‐tenth of the CsA dose typically required. Proteomic analysis verifies the protection mechanism, which includes reactive oxygen species inhibition, preservation of mitochondrial integrity, and reduced cellular apoptosis, suggesting potential for this biomimetic strategy in HIRI intervention.

## Graphical abstract

This work develops a biomimetic cyclosporin A (CsA) delivery system targeting hepatic lesion sites by constructing mesenchymal stem cell membrane‐camouflaged liposomes. The constructed hybrid nanovesicles effectively reduce liver damage and restore liver function at a one‐tenth dose of free CsA, demonstrating the potential of the nanovesicles to address the current challenges of hepatic ischemia‐reperfusion injury intervention.

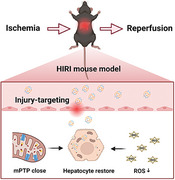

## 1 Introduction

Hepatic ischemia‐reperfusion injury (HIRI) is a gradually exacerbated phenomenon that arises during liver resection and transplantation when blood flow is reinstated following a period of interruption.^[^ ^1^ ^]^ This event leads to increased hepatocyte death due to adenosine triphosphate consumption and oxygen depletion, and triggers an inflammatory cascade and excessive production of reactive oxygen species (ROS), causing significant hepatocyte damage.^[^ ^2^ ^]^ These effects result in a cycle that increases the risk of early dysfunction and limits the availability of organs for transplantation.^[^ ^2^, ^3^ ^]^ Hence, identifying effective protection strategies to mitigate HIRI is a significant challenge in medical and public health fields.

Mitochondria, which are critical in maintaining cellular homeostasis, are implicated in the onset and progression of ischemic‐reperfusion injury (IRI).^[^ ^4^ ^]^ Initial mechanical damage disrupts cell membranes and cytoskeletal components, leading to increased intracellular calcium levels and activation of the mitochondrial permeability transition pore (mPTP). This results in mitochondrial swelling and rupture.^[^ ^5^ ^]^ Later, damaged mitochondria release cytochrome c and ROS, initiating cascade reactions that culminate in widespread apoptosis and inflammation in the liver.^[^ ^6^ ^]^ Given the crucial role of mitochondria, preserving or restoring mitochondrial homeostasis is a key objective in HIRI prevention.

Cyclosporine A (CsA), an immunosuppressant isolated from fungi, has been reported to pharmacologically inhibit the opening of mPTP.^[^ ^7^ ^]^ By binding with cyclophilin D (CypD) located in the inner mitochondrial membrane, CsA can reduce mitochondrial calcium uptake and ROS production, thereby alleviating mitochondrial dysfunction.^[^ ^8^ ^]^ Previous research has highlighted the potential of CsA in HIRI prevention.^[^ ^9^ ^]^ However, its poor aqueous solubility and high binding affinity to plasma proteins lead to low bioavailability and high‐dose requirements to achieve the desired protection effects.^[^ ^10^ ^]^ This, in turn, causes severe side effects such as immunosuppression, cytotoxicity, and oncogenesis, and significantly restricts its clinical application.^[^ ^11^ ^]^ In summary, the main challenge in CsA‐mediated HIRI prevention lies in achieving targeted delivery to the hepatic lesion sites while minimizing its adverse effects on healthy cells.

Nanovehicles like liposomes offer a promising drug delivery strategy due to their high drug‐loading capacity, sustained release profile, and biocompatibility.^[^ ^12^ ^]^ However, they face challenges such as rapid clearance by the mononuclear phagocyte system (MPS) and lack of selectivity between healthy and injured liver cells^[^ ^13^ ^]^ Natural cell membrane‐based nanomaterials, particularly the mesenchymal stem cells (MSCs), offer an exciting approach to overcome these challenges. Their low expression of major histocompatibility complex allows them to evade rapid clearance by immune cells.^[^ ^14^ ^]^ Meanwhile, their chemokine and adhesion molecule expression on their membrane enables targeted migration to injured tissues.^[^ ^15^ ^]^ These unique biological properties offer tremendous potential in addressing the challenges associated with drug delivery by liposomes, making them an attractive option for achieving biomimetic delivery of CsA in the field of HIRI prevention.

**Scheme 1:**
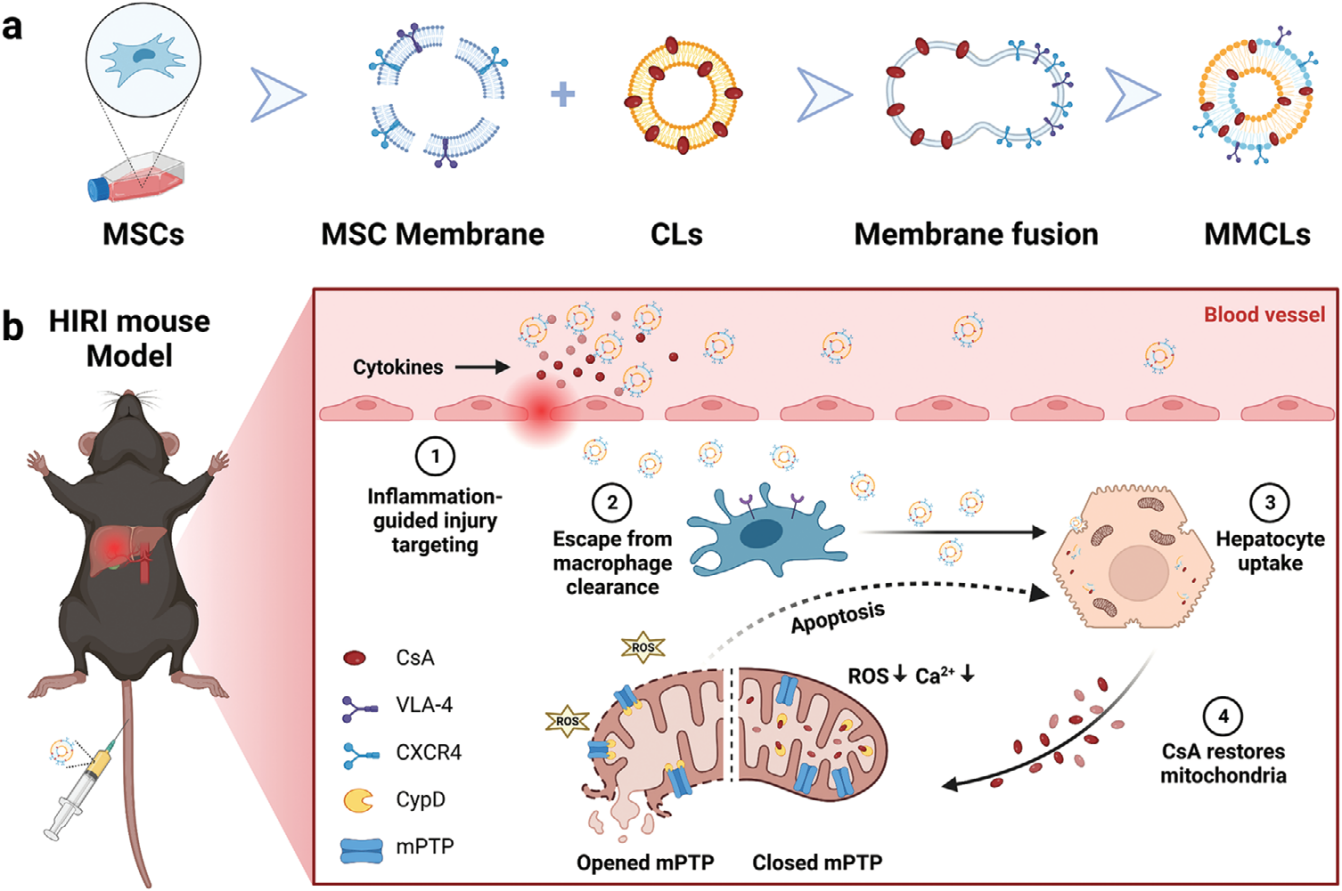
Schematic illustration of using biomimetic nanovesicles to prevent HIRI by MMs with CLs. a) Construction of MMCLs by the extrusion method. b) MMCLs exhibited inflammation chemotaxis to the hepatic lesion site and escaped from macrophage clearance after tail intravenous injection. Hepatocyte uptake of MMCLs can release CsA to bind with CypD on the mitochondrial membrane, close the mPTP, alleviate mitochondrial dysfunction, and reduce cell apoptosis.

In this study, we developed MSC membrane‐camouflaged CsA liposomes (MMCLs) for targeted HIRI intervention. By combining human umbilical cord‐derived MSC membrane with liposomal membrane, MMCLs were designed to retain the inflammatory chemotaxis and immune privilege of MSCs, along with efficient drug loading of liposomes (**Scheme** **1**). The fusion of membranes had minimal impact on the encapsulation capacity of CsA. In vitro and in vivo experiments demonstrated the superior ability of MMCLs to mitigate mitochondrial dysfunction, reduce ROS production, and decrease cell apoptosis. In a HIRI mouse model, the inflammatory microenvironment characterized by overexpression of pro‐inflammatory cytokines and chemokines at the hepatic lesion sites provided a suitable target. The biomimetic vesicles, MMCLs, exhibited efficient accumulation in the injured liver and successfully evaded clearance by the MPS in the liver and spleen. Benefiting from targeted delivery, MMCLs significantly reduced liver damage and restored liver function at a one‐tenth dose‐free CsA. Importantly, MMCLs mitigated the side effects of the drugs on healthy organs and cells, presenting a promising protection option for HIRI.

## 2 Results and Discussion

### 2.1 Construction and Characterization of the Hybridized NVs

**Figure 1:**
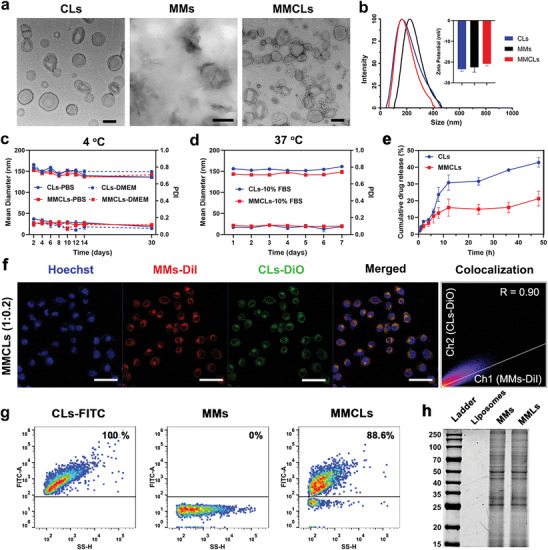
Characterization of CLs, MMs, and MMCLs. a) Morphology of CLs, MMs, and MMCLs under TEM. b) DLS size distribution and zeta potentials of CLs, MMs, and MMCLs. c) Time‐dependent colloidal stability and dispersibility of CLs and MMCLs in PBS or DMEM at 4 °C for 30 days. d) Time‐dependent stability and dispersibility of CLs and MMCLs in 10% FBS at 37 °C for 7 days. e) Drug release profile of CLs and MMCLs in PBS buffer containing 0.5% Tween 80 under 37 °C for 48 h. f) Colocalization analysis of CLs and MMs after co‐extruded at phospholipid‐to‐membrane protein ratio of 1:0.2 and incubated with AML12 cells for 2 h. CLs were labeled by DiO and MMs were labeled by DiI, as visualized using a CLSM. Scale bar: 50 µm. g) The FITC‐positive ratio of CLs‐FITC, MMs, and MMCLs after co‐extruded at phospholipid‐to‐membrane protein ratio of 1:0.2 as detected by nano‐flow cytometry. h) The protein profile of liposomes, MMs, and MMLs as measured by SDS‐PAGE.

The preparation of MMCLs involved three steps: synthesis of CsA liposomes (CLs), isolation of MSC membranes (MMs), and camouflaging CLs with MSC membranes (Scheme 1a). CLs were first synthesized using a thin‐film hydration method. The encapsulation efficiency and loading capacity of CsA in CLs were determined to be 98.0% ± 2.8% and 4.8 ± 0.1%, respectively by high‐performance liquid chromatography (HPLC). MMs were obtained following a previously reported method.^[^ ^16^ ^]^ The collected MMs did not exist nuclei, as verified by prestaining the cell nuclei using DAPI and inverted fluorescence microscopic images (Figure S1, Supporting Information). The protein content in the purified MMs obtained from 3 × 10^7^ cells was determined to be 330 µg. Subsequently, the purified MMs were sonicated, mixed with CLs, and extruded through a series of polycarbonate membranes with pore sizes of 400 and 200 nm to produce MMCLs. Various physicochemical characterizations were performed as follows. HPLC detected only 2% of CsA leakage in MMCLs, indicating that the membrane fusion step had no significant effect on the loading capacity of CsA. Transmission electron microscopy (TEM) images revealed that the CLs presented smooth and spherical surfaces with a particle size of 100–200 nm, which was consistent with the morphology of large unilocular liposomes reported before.^[^ ^17^ ^]^ After co‐extrusion with wrinkled‐surface MMs, the resulting MMCLs maintained a smooth spherical structure on their surface with no multilamellar vesicles inside (**Figure** **1**a), proving that CLs and MMs were fused through the outer membrane rather than encapsulated. Therefore, CsA as a hydrophobic drug should be located within the phospholipid bilayer of the CLs and MMCLs, rather than in the aqueous core. Dynamic lighting scattering (DLS) measurements showed that the hydrodynamic diameter of MMCLs (152.8 ± 1.5 nm) was smaller than that of MMs (217.1 ± 4.0 nm) and similar to that of CLs (158.4 ± 1.4 nm), indicating the successful reconstruction of the two types of NVs through co‐extrusion. The zeta potentials of CLs, MMs, and MMCLs were all ≈−20 mV (Figure 1b).

To assess the stability of the NVs, CLs, and MMCLs were dispersed in different solutions and monitored for prolonged periods. As shown in Figure 1c, the particle size of CLs and MMCLs slightly decreased from 156 to 140 nm after 30 days of storage at 4 °C in phosphate buffer solution (PBS) or Dulbecco's modified Eagle's medium (DMEM), while the polydispersity index (PDI) remained consistently below 0.3, demonstrating their good stability and dispersibility. Also, the suspensions of CLs and MMCLs exhibited a strong Tyndall effect^[^ ^18^ ^]^ and showed no visible precipitation after 30 days of storage (Figure S2, Supporting Information), confirming the colloidal stability of the NVs. Furthermore, the physiological temperature might impact the structure of NVs, and serum proteins that nonspecifically adhere to the surface of nanoparticles might lead to tacrophage clearance. After storage in 10% fetal bovine serum (FBS) at 37 °C for 7 days, CLs and MMCLs both maintained stable particle size and low PDI (Figure 1d), providing the potential for a prolonged blood circulation half‐life. The release pattern was also investigated in PBS buffer (pH 7.4 containing 0.5% Tween 80) at 37 °C. Both CLs and MMCLs showed 12% drug release within 6 h. Then 42% of CsA was released from CLs at 48 h, whereas 21% of CsA was released from MMCLs, indicating that cell membrane hybridizing delayed the drug release of MMCLs (Figure 1e).

To determine the decoration degree of MMs onto CLs, the colocalization between CLs and MMs was measured at different phospholipid‐to‐membrane protein weight ratios of 1:0.025, 1:0.05, and 1:0.2, respectively. Initially, CLs and MMs were labeled with two lipophilic dyes, 3,3′‐dioctadecyloxacarbocyanine perchlorate (DiO) and 1,1′‐dioctadecyl‐3,3,3′,3′‐tetramethyl‐indocarbocyanine perchlorate (DiI), respectively, defined as CLs‐DiO and MMs‐DiI. After co‐extrusion, all groups were incubated with alpha mouse liver 12 (AML12) for 2 h and observed using a confocal laser scanning microscope (CLSM). Obviously, as the proportion of MMs increased, there was stronger DiI fluorescence in cells (Figure 1f; Figure S3, Supporting Information). Analysis of Pearson's correlation coefficient (R) demonstrated that CLs‐DiO exhibited good colocalization with MMs‐DiI at a phospholipid‐to‐membrane protein weight ratio of 1:0.2, with an *R‐*value of 0.90, which was significantly higher than at ratios of 1:0.05 (*R* = 0.71) and 1:0.025 (*R* = 0.55). Furthermore, to determine the efficiency of membrane fusion, CLs labeled with DSPE‐PEG‐FITC (CLs‐FITC) were co‐extruded with non‐labeled MMs at a ratio of 1:0.2 to produce MMCLs. Nano‐flow cytometry analysis revealed that the FITC‐positive ratio of CLs‐FITC was 100%. After fusion with non‐fluorescent MMs, the FITC‐positive ratio of MMCLs decreased to 88.6% (Figure 1g). These results indicated that ≈90% MMs were fully fused with CLs in the presence of a phospholipid‐to‐membrane protein weight ratio of 1:0.2, which was determined as the appropriate ratio for MMCLs preparation. Considering the crucial role of proteins derived from MSCs, the protein remaining on the hybrid NVs was further confirmed using sodium dodecyl sulfate‐polyacrylamide gel electrophoresis (SDS‐PAGE). Compared with empty liposomes without protein expression, the protein profiles of MSC membrane‐camouflaged liposomes (MMLs) were completely consistent with that of MMs, demonstrating that the total protein profiles of MMs were preserved after co‐extrusion (Figure 1h). These findings provided strong evidence for the successful decoration of CLs with the phospholipid structure and protein profile of MMs, which is expected to impart biomimetic properties to MMCLs.

### 2.2 Inflammatory Chemotaxis and Endocytosis Pathway of NVs

**Figure 2:**
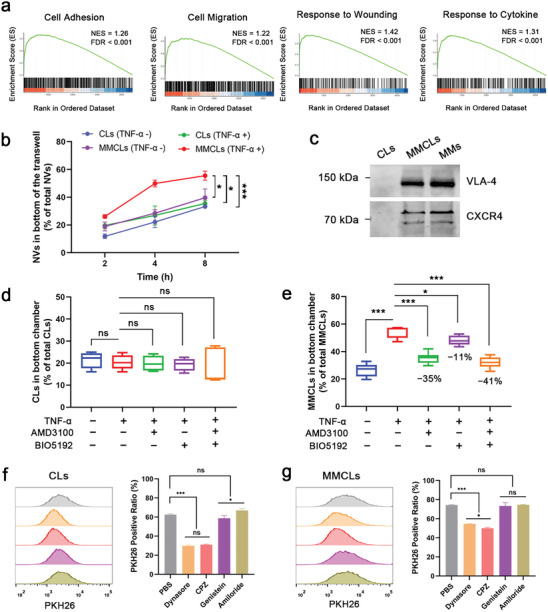
Inflammatory chemotaxis and endocytosis pathway of CLs and MMCLs. a) Gene Set Enrichment Analysis (GSEA) of MMs. Enrichment plots for significant datasets enriched in GSEA analysis show the profile of the normalized ES and false discovery rate (FDR) ratio. b) The amount of NVs in the bottom chamber at 2, 4, and 8 h after the addition of PKH26‐labeled CLs or MMCLs (*n* = 3). c) CXCR4 and VLA‐4 expression of CLs, MMCLs, and MMs detected by western blot assay. Inhibition by AMD3100 and BIO5192 of the suppression of transendothelial migration capability of PKH26‐labeled d) CLs and e) MMCLs (*n* = 5). Flow cytometry analysis and quantification of mean fluorescence intensity of PKH26‐labeled f) CLs and g) MMCLs internalized by AML12 cells under different endocytosis inhibitors (*n* = 3). Data are presented as mean ± SD. The statistical significance was analyzed using one‐way ANOVA following Tukey's multiple comparisons test (*p < 0.05; **p < 0.01; ***p < 0.001, ns = no significance).

The innate immune plays a crucial role in response to cellular injury during HIRI. Among these, pro‐inflammatory cytokines such as tumor necrosis factor (TNF‐α) contribute to the activation of endothelial cells, leading to increased permeability of the endothelium and the recruitment and activation of leukocytes.^[^ ^19^ ^]^ It has been observed that MSCs can also migrate through gaps and pores in the activated endothelial cells during inflammation.^[^ ^20^ ^]^ To explore the role of MMs in MSCs migration, we used liquid chromatography‐tandem mass spectrometry (LC‐MS/MS) to perform protein profiling of MMs and identified a total of 4456 high‐quality proteins. Gene Set Enrichment Analysis (GSEA) showed the most highly enriched categories in MMs included “Cell Adhesion,” “Cell Migration,” “Response to Wounding,” and “Response to Cytokine” (**Figure** **2**a; Figure S4, Supporting Information), indicating that a series of membrane proteins were involved in the inflammatory chemotaxis process of MSCs to the injury site. To confirm whether MMCLs decorated with MMs possess similar properties to MSCs, human umbilical vein endothelial cells (HUVEC) were cultivated in the upper chamber of a Transwell to form an in vitro model of transendothelial migration. TNF‐α was added in the bottom chamber to simulate inflammation‐activated HUVEC, and the mRNA expression was assessed before and after TNF‐α treatment. The addition of TNF‐α significantly increased the levels of adhesion molecules, such as intercellular adhesion molecule‐1 (ICAM‐1) and vascular cell adhesion protein 1 (VCAM‐1), as well as the pro‐inflammatory cytokine interleukin‐1β (IL‐1β), while decreasing the level of the anti‐inflammatory cytokine interleukin‐10 (IL‐10) (Figure S5, Supporting Information), confirming the successful activation of HUVEC.^[^ ^20^, ^21^ ^]^ Whereafter, the culture medium in the upper chamber was replaced with fresh medium containing PKH26‐labeled CLs or MMCLs. As demonstrated in Figure 2b, the transmigration efficiency of CLs, without MMs modification, showed no significant difference in the presence or absence of TNF‐α, indicating that cytokines did not affect their penetration behavior. In contrast, the number of MMCLs that crossed the endothelial layer in the TNF‐α‐treated (TNF‐α +) group was significantly higher compared to the TNF‐α‐untreated (TNF‐α −) group and the CLs groups. Similarly, the uptake of MMCLs was significantly more than that of CLs by hepatocytes cultured in the bottom chamber, 1.8 and 2.9 times higher at 2 and 4 h, respectively (Figure S6, Supporting Information). These observations verified the inflammatory chemotaxis and transendothelial migration properties of MMCLs, which were primarily attributed to the decorated with MMs containing chemokine receptors and adhesion molecules.

Among the membrane proteins, previous studies have shown that C‐X‐C chemokine receptor type 4 (CXCR4) and very late antigen‐4 (VLA‐4) are classical chemokine receptors and adhesion molecules expressed on MMs. The onset of inflammation in injured tissue causes the release of cytokines which upregulate VCAM‐1 and activate VLA‐4, leading to the initial arrest of MSCs on the endothelium surface.^[^ ^22^ ^]^ Thereafter, MSCs traverse through the endothelial cells and migrate toward injured sites following chemokine gradients, such as stromal cell‐derived factor 1α (SDF‐1α), mediated by the CXCR4/SDF‐1α pathway.^[^ ^23^ ^]^ Western blot images showed that the expression of VLA‐4 and CXCR4 in MMs was largely preserved during the preparation of MMCLs, but negative in CLs (Figure 2c). AMD3100, an inhibitor of CXCR4,^[^ ^24^ ^]^ and BIO5192, an inhibitor of VLA‐4,^[^ ^25^ ^]^ were applied in the in vitro model of transendothelial migration to verify their effective role in inflammatory chemotaxis. As demonstrated in Figure 2d, the addition of TNF‐α and inhibitors AMD3100 and BIO5192 had no significant effect on the transendothelial ability of CLs, which was ≈20%. Conversely, within the inflammatory environment, AMD3100 and BIO5192 independently decreased the transendothelial migration of MMCLs by 35% and 11%, respectively, and collectively reduced migration by 41% when combined (Figure 2e). These results proved that chemokine receptors represented by CXCR4, and adhesion molecules represented by VLA‐4 play an important role in inflammatory chemotaxis and transendothelial migration properties of MMCLs, which can enable them to target injured liver cells within the bloodstream more rapidly.

Furthermore, various chemical inhibitors were applied to investigate the endocytic pathways of CLs and MMCLs by flow cytometry analysis. As shown in Figure 2f,g, the cellular uptake of both NVs was significantly inhibited by dynasore, which is a dynamin‐dependent endocytosis inhibitor related to caveolin‐dependent and clathrin‐mediated pathways.^[^ ^26^ ^]^ To separately elaborate the function of clathrin and caveolin in the endocytosis, chloropromazine (CPZ, clathrin‐mediated endocytosis inhibitor) and genistein (caveolae‐dependent endocytosis inhibitor) were used, and the quantification of fluorescence intensity hinted that the inhibition rate of CPZ was basically the same as dynasore, while genistein had no significant inhibition on cell uptake. The results revealed that the clathrin‐mediated pathway could be a domain route of CLs and MMCLs that entry into the cells whereas caveolin‐dependent mechanisms were not favored. In addition, there was also no significant inhibition of cell uptake by amiloride, which is a Na^+^/H^+^ pump‐related macropinocytosis inhibitor.^[^ ^27^ ^]^ Taken together, these results indicated that CLs and MMCLs followed the same internalization pathway, and clathrin‐mediated endocytosis played the most significant role in hepatocyte uptake.

### 2.3 Biodistribution and Pharmacokinetic Behavior of CsA‐loaded NVs in a HIRI Mouse Model

**Figure 3:**
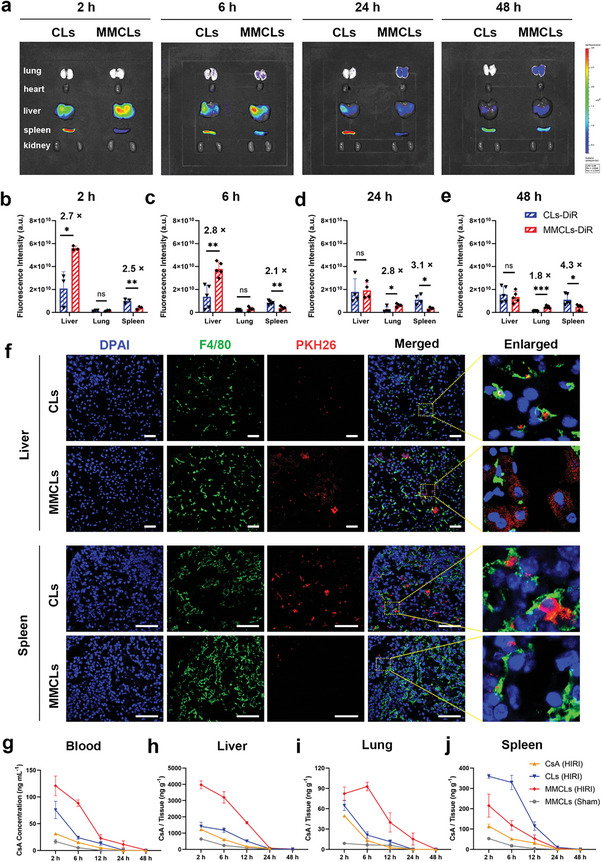
Biodistribution and pharmacokinetic behavior of CsA, CLs, and MMCLs in a HIRI mice model through tail vein injection. a) Ex vivo tissue distribution of DiR‐labeled CLs and MMCLs in the main organs at different time points and the corresponding fluorescence signals of liver, lung, and spleen at b) 2 h, c) 6 h, d) 24 h, and e) 48 h after reperfusion (data are analyzed using two‐tailed t‐test, *n* = 5, *p < 0.05; **p < 0.01; ***p < 0.001; and ns, no significant difference). f) Immunofluorescence images of liver and spleen sections after intravenous administration of PKH26‐labeled CLs or MMCLs, with cell nuclei stained blue (DAPI) and macrophages stained green (F4/80). Scale bar: 50 µm. Quantification of the concentration of CsA in g) blood, h) liver, i) lung, and j) spleen after injection of free CsA, CLs, or MMCLs (at a CsA dose of 0.1 mg kg^−1^) into HIRI mice or sham‐operated mice (*n* = 3). The samples were homogenized and subjected to quantification of CsA concentration through LC‐MS analysis.

A HIRI mouse model was established to evaluate the feasibility of a bionic membrane‐assisted delivery to the injured liver.^[^ ^28^ ^]^ To track the biodistribution of the NVs, the commercially available tracer 1,1′‐dioctadecyl‐3,3,3,3′‐tetramethyl‐indotricarbocyaine iodide (DiR) was used for labeling. It was determined that the supernatant after DiR labeling had no fluorescence, both in vivo and ex vivo, indicating that the free dye did not interfere with the imaging process (Figure S7, Supporting Information). Thereafter, 200 µL of DiR‐labeled CLs or MMCLs were injected into HIRI mice via the tail vein, and fluorescence imaging was conducted at various time points (2, 6, 24, and 48 h) following reperfusion. The results showed that MMCLs were initially enriched in the liver within 2 h after reperfusion and then decreased over time (**Figure** **3**a). Quantitative analysis of the ex vivo tissue fluorescence demonstrated a higher accumulation of MMCLs in the liver than the CLs group, with 2.7‐ and 2.8‐fold higher accumulation at 2 and 6 h, respectively (Figure 3b,c). Similarly, immunofluorescence images of liver sections taken 2 h after reperfusion revealed an increased number of MMCLs entering hepatocytes labeled with cytokeratin‐18 (CK18) antibody, in comparison to CLs (Figure S8, Supporting Information). These observations confirmed that MMCLs possessed a strong ability of chemotaxis toward the injured liver. Interestingly, as the fluorescence of MMCLs decreased in the liver over time, it gradually increased in the lung. After 24 and 48 h, the lung distribution of MMCLs was 2.8‐ and 1.8‐fold higher, respectively, compared to CLs (Figure 3d,e). Previous studies have revealed that the lung is the first organ to encounter reperfusion from the bloodstream following IRI in the liver. The increased oxidative stress and release of inflammatory cytokines play a critical role in mediating acute lung injury, which is a common postoperative complication after liver transplantation.^[^ ^29^ ^]^ Therefore, we hypothesized that the significantly higher distribution of MMCLs in the lung, in comparison to CLs, could be attributed to the inflammatory chemotaxis induced by MMs, which facilitated the NVs to penetrate the endothelium readily under inflammatory conditions.^[^ ^20^ ^]^ This hypothesis, however, warrants further investigation.

Conversely, CLs showed an abundant uptake by the spleen 2 h after reperfusion, which can be directly observed by in vivo fluorescence imaging (red arrows; Figure S9, Supporting Information). This tendency became even more pronounced at 24 and 48 h, reaching 3.1‐ and 4.3‐fold higher levels, respectively to MMCLs (Figure 3d,e). As reported previously, the liver and spleen both play important roles in immune surveillance and act as the blood‐cleansing device in the MPS.^[^ ^30^ ^]^ Opsonins present in the blood serum quickly bind to foreign materials, enabling macrophages to easily recognize and remove these nanoparticles.^[^ ^14^, ^31^ ^]^ Consequently, the accumulation of NVs in the macrophages of the spleen and liver (Kupffer cells) hinders the delivery of a sufficient dose of NVs to the destination.^[^ ^13^ ^]^ Therefore, the macrophages were stained with F4/80 antibody to investigate the detailed distribution of CLs and MMCLs in the liver and spleen sections. In the immunofluorescence images of liver and spleen sections (Figure 3f), the PKH26‐labeled CLs were completely colocalized with F4/80‐labeled cells, suggesting that the CLs were phagocytosed by the hepatic and splenic macrophages. Whereas MMCLs were hardly phagocytosed by Kupffer cells and splenic macrophages but instead entered the hepatocytes, and the overall fluorescence intensity of MMCLs in liver sections was much higher than that of CLs, which was consistent with ex vivo imaging. These results were attributed to the biomimetic functionalization of MMCLs with MMs, which disguised the MMCLs as stem cells, preventing recognition by macrophages, and thereby exhibiting a stronger ability to escape from the splenic and hepatic MPS.

To determine if the pharmacokinetic behavior and distribution of CsA were consistent with fluorescently labeled NVs, equivalent CsA dosage (0.1 mg kg^−1^) of free CsA, CLs, and MMCLs were injected into HIRI mice, and the samples of blood and major organs were collected at 2, 6, 12, 24, and 48 h after reperfusion. The samples were homogenized and subjected to quantification of CsA concentration through LC‐MS analysis. It was found that the higher and more constant CsA accumulation (120.8 ng mL^−1^ at 2 h and 88.6 ng mL^−1^ at 6 h) was achieved in the blood of the MMCLs‐treated HIRI mice compared to free CsA (31.5 ng mL^−1^ at 2 h) and CLs (75.7 ng mL^−1^ at 2 h and 24.1 ng mL^−1^ at 6 h). The elimination half‐life (*t*_1/2_) for CsA, CLs, and MMCLs in HIRI mice were 4.0, 4.1, and 7.6 h, respectively (Figure 3g). The prolonged blood circulation time could be attributed to the biomimetic functionalization of MMCLs with MMs, enhancing sustained drug release and reducing the clearance by MPS. In the IR‐injured liver, CsA accumulation in the MMCLs‐treated mice was 2.8‐ and 2.7‐fold higher than that of the CLs‐treated mice (Figure 3h), consistent with the fluorescence distribution of NVs. In contrast, the concentration of CsA in the liver of sham‐operated animals was negligible following the MMCLs treatment (Figure 3h). Moreover, CsA was specifically enriched in the lung at 6 h (Figure 3i). Together, these results demonstrated the inflammatory chemotaxis of MMCLs induced by MMs. In addition, the content of CsA in the spleen of MMCLs‐treated mice was lower than that of CLs‐treated mice (Figure 3j), which was related to the low immunogenicity of MMs. The dual capabilities of inflammation chemotaxis and macrophage escape dramatically improved the biodistribution of MMCLs in the liver of HIRI mice, concentrating them in the injured lesions. This enhanced biodistribution is expected to improve the bioavailability of drugs delivered by administrating MMCLs.

### 2.4 MMCLs Restored Mitochondrial Dysfunction in Hypoxia/Reoxygenation (H/R)‐Injured Hepatocytes

**Figure 4:**
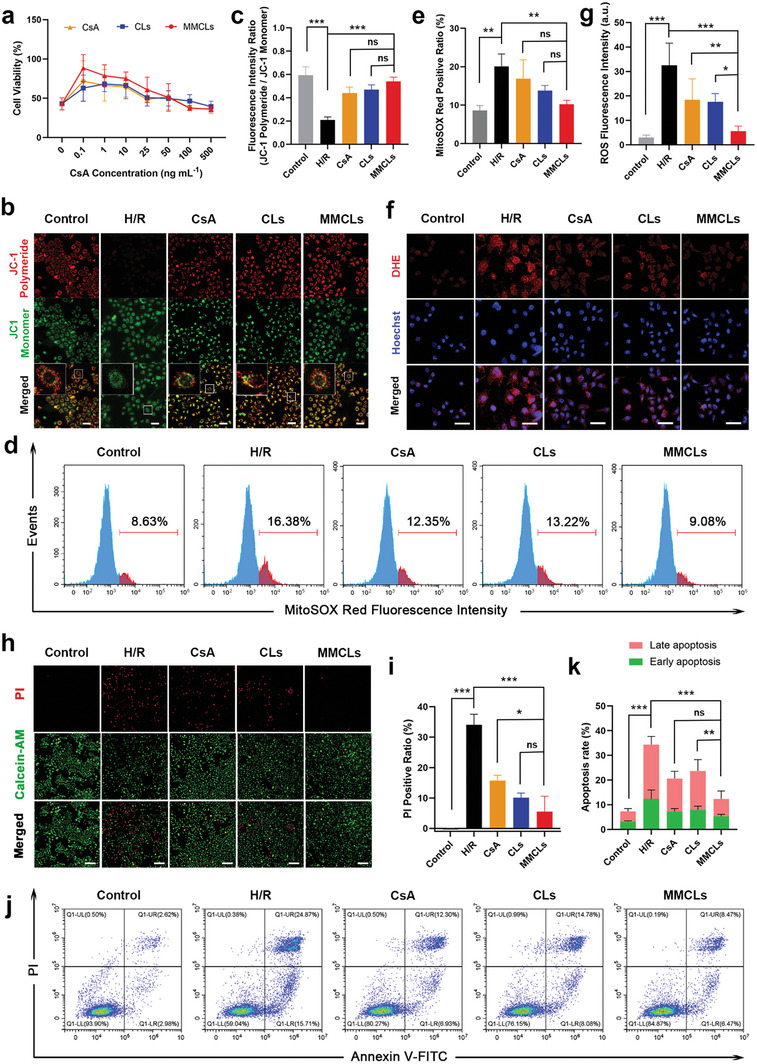
Verification of the protection effect of MMCLs on H/R‐injured AML12 cells in vitro. a) Cell viability of H/R‐injured cells after being treated with free CsA, CLs, and MMCLs at CsA concentrations of 0 − 500 ng mL^−1^ as evaluated by CCK‐8 assay. b) JC‐1 mitochondrial membrane potential in normal cells and H/R‐injured cells following the treatment with CsA, CLs, and MMCLs as imaged by CLSM (scale bar: 50 µm), and c) the corresponding fluorescence intensity ratio of JC‐1 polymeride and monomer (*n* = 5). d) Flow cytometry analysis of the superoxide levels using MitoSOX Red staining, and e) the corresponding positive ratio of MitoSOX Red (*n* = 5). f) CLSM images of AML12 cells stained by DHE (scale bar: 50 µm), and g) the corresponding fluorescence intensity of intracellular ROS (*n* = 5). h) HCI analysis of cell death using PI staining (scale bar: 200 µm) and i) the corresponding positive ratio of PI (*n* = 5). j) Flow cytometry analysis of early and late apoptosis by annexin V‐FITC/PI dual staining assay, and k) the corresponding rates of early and late apoptosis (*n* = 5). Data are presented as mean ± SD. The statistical significance was analyzed using one‐way ANOVA following Tukey's multiple comparisons test (*p < 0.05; **p < 0.01; ***p < 0.001, ns = no significance).

After confirming that injured hepatocytes efficiently take up MMCLs both in vitro and in vivo, we proceeded to study the protection potential of MMCLs in H/R‐injured AML12 cells. To assess the dose‐dependent response of drugs, we evaluated the viability of H/R‐pretreated AML12 cells using a cell counting kit‐8 (CCK‐8) after incubating with free CsA, CLs, or MMCLs at CsA concentrations ranging from 0 to 500 ng mL^−1^. As a control, AML12 cells cultured under normal conditions were used. Subjecting these cells to 8 h of hypoxia followed by 6 h of reoxygenation resulted in a significant decrease in cell viability to 42.9% (**Figure** **4**a). However, when treated with MMCLs at a CsA concentration of 0.1 ng mL^−1^, H/R‐induced cell death was rescued, and cell viability increased to 88.5%. This protective effect was superior to that of CsA or CLs at the same CsA concentration. The enhanced efficacy of MMCLs may be attributed to their efficient uptake by hepatocytes, which triggered the protection action of CsA during the early stages of mitochondrial damage. Furthermore, we observed that higher concentrations of CsA treatment led to a decrease in cell viability and exhibited minimal protective effects beyond 50 ng mL^−1^. This finding indicated that CsA not only possesses protection benefits but also exhibits significant cytotoxicity.^[^ ^32^ ^]^ This dose‐dependent response underscores the importance of controlling the CsA dosage to achieve the desired curative effect. Consequently, a CsA concentration of 0.1 ng mL^−1^ was selected for subsequent in vitro experiments to validate its efficacy.

First, we evaluated the mitochondrial function using JC‐1 mitochondrial membrane potential assay. An increase in the JC‐1 monomer signal indicates a decrease in mitochondrial membrane potential and early apoptosis of cells. AML12 cells were subjected to H/R treatment and then incubated with free CsA, CLs, or MMCLs for 4 h. The mitochondrial membrane potential after drug treatments was significantly higher than that in the H/R group, with MMCLs demonstrating a more pronounced effect in maintaining mitochondrial homeostasis compared to CsA and CLs (Figure 4b,c). Mitochondria are known as the primary site of superoxide generation, where various antioxidant systems maintain a balance under normal conditions but become insufficient during ischemia/reperfusion insult.^[^ ^33^ ^]^ Considering that excessive superoxide formation can lead to damage to mitochondrial DNA, proteins, and lipids, inhibition of superoxide overproduction is crucial for the suppression of IRI.^[^ ^34^ ^]^ Therefore, the levels of superoxide in the H/R‐injured cells were assessed using a mitochondrial superoxide indicator (MitoSOX Red) after treatment with free CsA, CLs, or MMCLs. Compared to the control group with a MitoSOX Red positive ratio of 8.63%, H/R treatment induced the production of large amounts of superoxide, resulting in a ratio of 16.38%. However, the addition of MMCLs significantly downregulated the superoxide level in H/R‐injured cells, reaching a MitoSOX Red level (9.08%) similar to that of the control group. This effect was distinct from the treatments with free CsA and CLs treatments (Figure 4d,e). Furthermore, the total levels of ROS in the different groups were also investigated using a dihydroethidium (DHE) assay kit. CLSM observations revealed that MMCLs significantly reduced the ROS level in the H/R‐injured cells compared to CsA and CLs (Figure 4f,g), effectively mitigating the damage to cellular activity caused by by‐products of mitochondrial dysfunction. These results demonstrated that MMCLs can efficiently maintain mitochondrial membrane potential and reduce the production of superoxide and ROS.

Building upon the aforementioned results, we further evaluated the reparative effect of MMCLs on injured cells using propidium iodide (PI) staining, visualized through a high‐content imaging (HCI) system. As shown in Figure 4h,i, with the dual regulation of mitochondrial function and ROS production, MMCLs reduced the positive rate of PI staining from 34.1% to 5.5% in H/R‐injured AML12 cells. This reduction was lower compared to the CsA (15.8%) and CLs (10.1%) treatment groups. Similarly, the annexin V‐FITC/PI dual staining assay demonstrated that the percentage of early and late apoptosis was the lowest in the MMCLs group, confirming its superior protection effect on H/R injury (Figure 4j,k). However, in the group treated with an equivalent protein dose of MMs, the cell viability and total apoptosis rate of the injured hepatocytes did not significantly change, indicating that the independent effect of MMs on H/R injury can be considered negligible (Figure S10, Supporting Information). Overall, MMCLs effectively mitigated mitochondrial dysfunction and ROS production caused by H/R treatment, leading to a significant reduction in apoptotic hepatocytes and contributing to the improved recovery of hepatic function in HIRI.

### 2.5 MMCLs Effectively Alleviated the Hepatic Injury In Vivo

**Figure 5:**
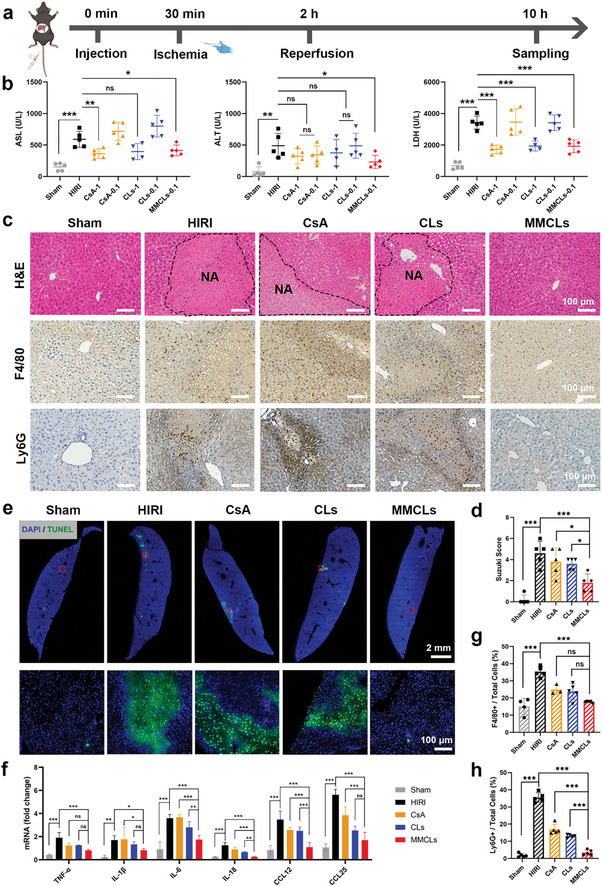
Alleviation of the hepatic injury by CsA, CLs, and MMCLs in a HIRI mouse model. a) Schematic illustration for the drug treatments in HIRI mice. C57BL/6 mice were intravenously injected with PBS, CsA, CLs, or MMCLs, followed by an I/R procedure, and then the blood and liver samples were collected 10 h after injection. A sham‐operated group of healthy mice (Sham) was set as a control. b) Evaluation of liver functions of AST, ALT, and LDH after administration of PBS, CsA, CLs, or MMCLs at a CsA dosage of 0.1 or 1 mg kg^−1^ (*n* = 5). c) H&E staining and immunohistochemical staining of liver sections in different treatment groups. NA, necrotic area. Macrophages were stained using F4/80 and neutrophils using Ly6G. d) Suzuki scores of the H&E staining (*n* = 5). e) TUNEL images visualized by a panoramic scanning microscope and the enlarged images of the red boxes. f) The mRNA expression of inflammatory cytokines and chemokines in hepatic tissues was measured by RT‐qPCR (*n* = 5). Percentage of g) macrophages (F4/80+) and h) neutrophils (Ly6G+) in total cells of liver sections after the indicated treatments (*n* = 5). Data are presented as mean ± SD. The statistical significance was analyzed using one‐way ANOVA following Tukey's multiple comparisons test (*p < 0.05; **p < 0.01; ***p < 0.001, ns = no significance).

Subsequently, the protection efficacy of free CsA, CLs, and MMCLs was compared in a HIRI mouse model. The experiment involved different groups, including a sham‐operated group of healthy mice (Sham) as a control. As depicted in **Figure** **5**a, the experimental groups involved intravenous injection of PBS, CsA, CLs, or MMCLs into the mice. An atraumatic clip was used to interrupt the blood supply of liver lobes for 90 min to simulate an ischemic period, and then blood and liver samples were collected after 8 h of reperfusion to evaluate liver function. The levels of aspartate aminotransferase (AST), alanine aminotransferase (ALT), and lactate dehydrogenase (LDH) were measured. Increased levels of these indicators are direct indications of liver injury.^[^ ^35^ ^]^ The results showed that the HIRI mice injected with PBS (HIRI group) exhibited significant elevations in AST, ALT, and LDH levels compared to the Sham group (Figure 5b), confirming liver damage caused by the ischemia/reperfusion surgery. Then, the protective dose of CsA and MMCLs was optimized (Figure S11, Supporting Information), showing that treated with CsA or CLs at a dosage of 1 mg kg^−1^ (CsA‐1 or CLs‐1) most effectively reduced AST, ALT, and LDH levels, but lower dosage of 0.1 mg kg^−1^ of CsA (CsA‐0.1) and CLs (CLs‐0.1) did not show any effect on these indicator levels. In comparison, treatment with MMCLs at a low CsA dosage of 0.1 mg kg^−1^ (MMCLs‐0.1) significantly down‐regulated the three hepatic enzymes, despite the injection amount being only 1/10 of the effective dosage used for CsA and CLs (1 mg kg^−1^). To exclude the influence of MMs on liver injury, liver function indicators were measured in HIRI mice that were solely injected with MMs at a protein dosage equivalent to MMCLs‐0.1. It was found that AST and LDH levels of the MMs‐treated group did not change significantly, while liver sections injected with MMs exhibited large areas of hepatocyte necrosis (Figure S12, Supporting Information), indicating that the independent effect of MMs on liver injury can be considered negligible. Previous studies have shown that hepatocyte membrane injury, such as lipid peroxidation induced by ROS, leads to increased membrane permeability and the release of hepatic enzymes like ALT into the bloodstream.^[^ ^36^ ^]^ Prolonged hepatic injury may result in necrosis and subsequent liberation of mitochondrial AST into the blood.^[^ ^37^ ^]^ Moreover, LDH production was increased under hypoxic conditions, enabling cells to generate adenosine triphosphate and maintain viability in a low‐oxygen environment.^[^ ^38^ ^]^ This evidence collectively demonstrated the potential of MMCLs administration at a relatively low dosage of CsA in effectively restoring liver function, including improving cell membrane permeability, maintaining mitochondrial homeostasis, and enhancing energy metabolism in mice with HIRI.

Due to the significant impact of HIRI on liver dysfunction and subsequent cell death, the extent of hepatocyte necrosis in the vicinity of liver lesions was assessed using hematoxylin−eosin (H&E) staining. Necrotic areas (NA), characterized by apoptotic and dead cells with crumbled nuclei and reddish cytoplasm, were visually distinguishable from healthy cells by a distinct boundary (black dashed line). To ensure a fair comparison of protection effects, all subsequent experiments were conducted using a consistent CsA dosage of 0.1 mg kg^−1^ across CsA, CLs, and MMCLs groups. Liver sections from HIRI mice injected with PBS, CsA, and CLs exhibited significant areas of hepatocyte necrosis (Figure 5c). However, the administration of MMCLs markedly ameliorated liver damage with fewer areas of hepatocyte necrosis and a clear reduction in the Suzuki score (1.8 ± 0.8) compared to the HIRI group (4.8 ± 1.1) (Figure 5d). Also, a terminal deoxynucleotidyl transferase (TdT)‐mediated dUTP nick end labeling (TUNEL) assay was employed to demonstrate the overall impact of different drug treatments on the proportion of apoptosis cells labeled green (Figure 5e), indicating that MMCLs most effectively mitigate necrosis and apoptosis of hepatocytes in HIRI at a low dosage of 0.1 mg kg^−1^.

In fact, HIRI is a local sterile inflammatory response driven by innate immunity.^[^ ^39^ ^]^ Assessing the inflammatory immune response of the liver before and after drug intervention is critical to understanding the occurrence and progression of liver damage. Therefore, the expression of inflammatory cytokines and chemokines, as well as activation and recruitment of macrophages and neutrophils in liver tissue were evaluated. As shown in Figure 5f, the mRNA levels of pro‐inflammatory cytokines (TNF‐α, tumor necrosis factor‐α; IL‐1β, interleukin‐1β; IL‐6, interleukin‐6; IL‐18, interleukin‐18) and chemokines (CCL12, C‐C chemokine ligand 12; CCL25, C‐C chemokine ligand 25) were strikingly upregulated after IR injury. The macrophage (F4/80+) and neutrophil (Ly6G+) infiltration were investigated by immunohistochemical staining, showing a marked increase in macrophages and neutrophils in liver sections taken from the HIRI group compared to the Sham group (Figure 5c). According to previous research, injured hepatic cells release damage‐associated molecular patterns (DAMPs), which can activate macrophages (Kupffer cells) that in turn secrete inflammatory cytokines. This contributes to further hepatocyte injury and the release of chemokines, which promote the recruitment of C–C motif chemokine receptor 2+ (CCR2+) neutrophils into the IR‐stressed liver and trigger parenchymal cell damage.^[^ ^40^ ^]^ In this work, treatment of MMCLs significantly lowered TNF‐α, IL‐1β, IL‐6, and IL‐18 expression, revealing the most effective anti‐inflammatory function compared to CsA and CLs (Figure 5f). Concomitantly, MMCLs strikingly decreased the expression of chemokines and inflammatory cell infiltration, where the number of macrophages and neutrophils in liver sections decreased by 49% and 90%, respectively (Figure 5g,h). These results demonstrated that MMCLs can alleviate IR‐induced liver damage by attenuating the innate inflammatory response.

Considering the importance of the biosafety of drugs in clinical application, histomorphological observations and functional assessments of major organs were performed in healthy mice after injection with CsA, CLs, or MMCLs at their effective protective dosage. Notably, no significant changes in cellular morphology were observed in the liver, kidney, heart, and lung following treatment with different drugs (Figure S13, Supporting Information). However, AST and ALT of liver function indicators, creatinine of kidney function indicators, as well as creatine kinase (CK) of heart function indicators were significantly higher than that of healthy mice with PBS after administration of CsA (Figure S14, Supporting Information). Also, AST, ALT, CK, and CK‐MB values were increased in the CLs‐treated group, indicating that the CsA dose of 1 mg kg^−1^ presented liver, kidney, and cardiac toxicity to some extent. In comparison, there was no significant change in all the above indicators in the MMCLs‐treated group, proving the superior biosafety at a protective dose of 0.1 mg kg^−1^ of MMCLs. Consequently, MMCLs hold significant potential as a highly effective delivery system, capable of minimizing side effects, widening the treatment window, and enhancing the clinical applicability of CsA in HIRI.

### 2.6 Protection Mechanism of MMCLs on HIRI Mice

**Figure 6:**
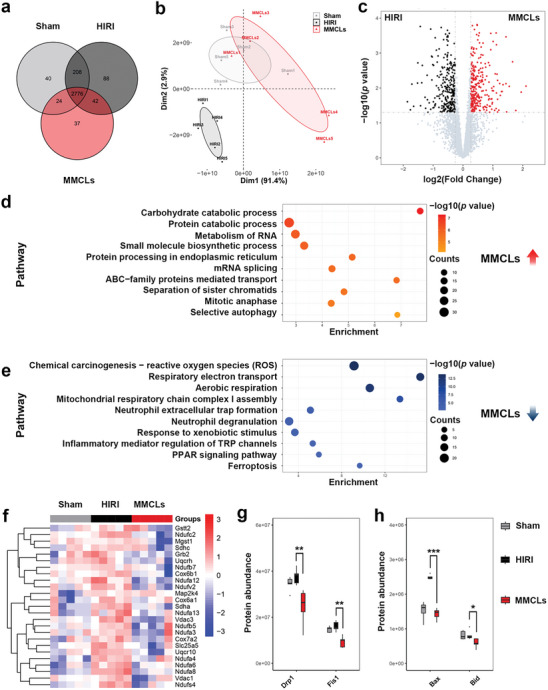
Protein profile analysis of liver samples from the Sham group, HIRI group, and MMCLs treatment group. a) Venn diagram showing the overlap of high‐quality proteins. b) Principal components analysis of the enriched proteins. c) Volcano diagram showing the differently expressed proteins between the HIRI and the MMCLs treatment group, fold change > 1.2 or < 0.8, *p* < 0.05. Functional enrichment analysis of the differently expressed proteins d) upregulated or e) downregulated in the MMCLs treatment group compared to the HIRI group. f) Heatmap generated by clustering of the differentially expressed proteins in the three groups. Red: up‐regulation; blue: downregulation. The expression quantity of the corresponding proteins enriched in positive regulation of g) mitochondrial fission and h) cellular apoptosis. Data are analyzed using a two‐tailed *t*‐test, *p < 0.05; **p < 0.01; ***p < 0.001.

Proteomic analysis of mouse liver tissues was performed to investigate the molecular mechanisms of MMCLs treatment on HIRI. Liver tissues from the Sham group, HIRI group, and MMCLs treatment group were analyzed using LC‐MS/MS, identifying a total of 3215 high‐quality proteins. Among these 2776 proteins were detectable in all three groups (**Figure** **6**a). Principal component analysis revealed significant differences in protein expression between the HIRI group and the other groups, while the protein expression profile of the MMCLs treatment group closely resembled that of the Sham group (Figure 6b). These findings suggested a notable influence of IRI on hepatic protein expression, which was effectively attenuated by the MMCLs treatment.

Differential protein expression analysis identified 126 differentially expressed proteins between the HIRI and Sham groups, encompassing 70 upregulated and 56 downregulated proteins (Figure S15, Supporting Information). Through pathway enrichment analyses, it was found that in the context of HIRI, an upregulation occurred in pathways such as “respiratory electron transport,” “ROS generation,” “mitochondrial organization,” “ferroptosis,” “apoptotic mitochondrial changes,” and “regulation of myeloid leukocyte mediated immunity” (Figure S16a, Supporting Information). In contrast, pathways related to “autophagy,” “steroid metabolic process,” “mRNA splicing,” “biosynthesis of nucleotides sugars,” “negative regulation of MAPK pathway,” and “IL‐6 signaling pathway” exhibited significant downregulation (Figure S16b, Supporting Information). These results confirmed that during HIRI, oxidative stress, cell apoptosis, and inflammatory responses were activated, while cellular metabolism and biosynthesis were suppressed, which was consistent with previous research findings.^[^ ^41^ ^]^

Comparing the MMCLs treatment group with the HIRI group, 548 differentially expressed proteins were identified, of which 272 were upregulated and 276 were downregulated (Figure 6c). Pathway enrichment analysis revealed that MMCLs treatment group upregulated pathways related to “carbohydrate catabolic processes,” “protein catabolic processes,” “RNA metabolism,” “mRNA splicing,” “small molecule biosynthetic processes,” “sister chromatid separation,” and “mitotic anaphase” (Figure 6d). Conversely, pathways related to “ROS,” “respiratory electron transport,” “assembly of mitochondrial respiratory chain complex I,” “formation of neutrophil extracellular traps,” “neutrophil degranulation,” “PPAR signaling pathways,” and “ferroptosis” were found to be downregulated (Figure 6e). Among these, the generation of ROS was observed to be the most prominently downregulated pathway.

Elevated levels of proteins associated with the ROS signaling pathway in the HIRI group were mitigated following MMCLs treatment (Figure 6f). The majority of these proteins constitute the mitochondrial electron transport chain,^[^ ^42^ ^]^ the primary locus for ROS generation in IRI.^[^ ^34^, ^43^ ^]^ Elevated ROS levels can induce mitochondrial oxidative stress leading to mitochondrial damage characterized by excessive fission and abnormal function.^[^ ^44^ ^]^ In the HIRI group, proteins like Drp1 and Fis1, which regulate mitochondrial fission,^[^ ^45^ ^]^ exhibited increased expression, but this was attenuated following MMCLs treatment (Figure 6g). Likewise, the expression of Bax and Bid, proteins implicated in apoptosis,^[^ ^46^ ^]^ was diminished in the MMCLs treatment group (Figure 6h). These results convincingly demonstrated that MMCLs treatment substantially inhibited the generation of mitochondrial ROS subsequent to HIRI, thereby preserving mitochondrial integrity by reducing mitochondrial fission and alleviating subsequent cellular apoptosis. In summary, these findings provided invaluable insights into the molecular mechanisms underlying HIRI and underscored the protection potential of MMCLs.

## 3 Conclusion

Here, we developed biomimetic nanovesicles by hybridizing liposomes with MSC membranes for targeted CsA delivery to achieve early intervention in HIRI. By membrane fusion, MMCLs exhibited inflammatory chemotaxis and immune privilege due to the inherited CXCR4 and VLA‐4 expression from MSC membranes, along with efficient drug loading of liposomes. Upon in vivo study, MMCLs accumulated in the injured liver at a level 2.7 times higher than that of liposomes, and conversely 2.5 times less engulfed by splenic macrophages than liposomes. This biomimetic delivery system remarkably reduced liver damage and restore function at a 1/10 dose of free CsA in a HIRI mouse model, with no significant side effects. Proteomic analysis revealed the protection mechanism of MMCLs, including the downregulation of the proteins associated with ROS production, reducing mitochondrial fission to maintain mitochondrial integrity, and ultimately alleviating cellular apoptosis. These findings provide strong evidence supporting the potential of MMCLs as an efficient protection option for HIRI prevention and highlight the versatility of MSC‐mediated biomimetic nanovesicles as a flexible nanoplatform for injury‐targeted drug delivery in vivo.

## 4 Experimental Section

### Culture of Cell Lines

AML12 cells and HUVEC were purchased from Guangzhou Jennio Biotech Co.,Ltd (Guangzhou, China). AML12 cells were cultured in DMEM: F12 medium supplemented with 10% FBS, 10 µg mL^−1^ insulin, 5.5 µg mL^−1^ transferrin, 5 ng mL^−1^ selenium, and 40 ng mL^−1^ dexamethasone. HUVEC were incubated in a commercial endothelial cell medium (ScienCell Research Laboratories, Inc., USA). All cells were cultured at 37 °C in a humidified 5% CO_2_ incubator.

### Isolation and Culture of UC‐MSCs

The procedures for isolating UC‐MSCs have been proved by the Human Research Ethics Committee of the Third Affiliated Hospital of Sun Yat‐sen University. UC‐MSCs were isolated and cultured under aseptic maintenance. Umbilical cords were collected after the donor agreed to sign the consent form and washed twice using PBS to wipe out the remnant blood. The umbilical cords were cut into 10 mm^3^ piece^−1^ and placed in type I collagenase containing hyaluronidase (0.1%) and CaCl_2_ (3 mm). After 4 h digestion at 37 °C, the umbilical cords were transferred into low‐glucose (1 g L^−1^) DMEM basic medium with 10% FBS and cultured in the humidified atmosphere with 5% CO_2_ at 37 °C. Medium was refreshed every 3 days to remove nonadherent cells.

### Membrane Derivation

Briefly, umbilical cord MSCs were grown to 80% confluence in multiple T‐225 culture flasks and harvested, followed by washing in 1× PBS thrice by centrifuging at 500 × g for 5 min. The purified cells underwent freezing and thawing repeatedly from −20 to 37 °C three times, followed by getting enucleated in a hand‐held Dounce homogenizer (20 passes while on ice). After centrifugation at 3200 × g for 5 min at 4 °C, the supernatant was gathered and centrifuged at 15 000 × g for 30 min for membrane collection. The membrane fragments were ultrasonicated (100 W) for 2 min and stored in 1× PBS at −80 °C for subsequent experiments. The protein content in the purified MSC membranes was determined by a bicinchoninic acid (BCA) protein quantification kit (Beyotime, China) for further preparation of MMCLs.

### Preparation and Characterization of CLs

CLs were prepared by a thin‐film hydration method. Phosphatidylcholine (Ruixi, Xi'an, China), cholesterol (Sigma–Aldrich, USA), and CsA (MedChemExpress, USA) (20:2.5:1, w/w) were dissolved in chloroform and evaporated to form a thin film in a round flask. Subsequently, the film was hydrated with 1× PBS at 40 °C for 30 min with a CsA concentration of 0.5 mg mL^−1^. Then, they were extruded with a Mini‐Extruder (Avanti Polar Lipids, Inc., USA) using a polycarbonate membrane with a pore size of 200 nm for ten cycles to develop CLs. To remove the free CsA, CLs were placed in a dialysis bag (MWCO = 3500), submerged in 1× PBS, and stirred at 100 rpm at 4 °C for 48 h.

### Preparation and Characterization of MMCLs

To synthesize MMCLs, a modified extrusion approach was used according to a previous report.^[^ ^47^ ^]^ Briefly, CLs were mixed with the purified MSC membranes at the phospholipid‐to‐membrane protein weight ratios of 1:0.025, 1:0.05, or 1:0.2, and then extruded through a polycarbonate membrane with pore sizes of 400 and 200 nm. To remove the free CsA, MMCLs were placed in a dialysis bag (MWCO = 3500), submerged in 1× PBS, and stirred at 100 rpm at 4 °C for 48 h. Particle size and zeta‐potential of CLs, MSC membranes, and MMCLs were measured by DLS using an EliteSizer (Brookhaven, USA), and the morphology was observed under a TEM (Talos L120C, ThermoFisher, USA) following uranyl acetate (UA) staining.

### Drug Loading and Release

To determine the encapsulation efficiency (EE) and loading capacity (LC) of CsA in CLs, free CsA was separated by centrifugation at 8 000 × g for 15 min with an ultrafiltration tube (100 000 Da, Millipore). The drug amount was quantified using an HPLC system (Waters 1525, USA) under the following conditions: column, ZORBAX SB‐C18 column (250 × 4.6 mm, 5 µm, Agilent); mobile phase, acetonitrile−methanol (80:20, v/v); flow rate, 1.0 mL min^−1^; detection wavelength, 210 nm; column temperature, 70 °C. The EE and LC were calculated by the following equations:

(1) $$\mathrm{EE}\;\left(\%\right)=\left(1-\frac{C\;\times \;n\;\times \;V}{T}\right)\;\times 100\%$$

(2) $$\mathrm{LC}\;\left(\%\right)=\frac{T\;-\;C\;\times \;n\;\times \;V}{W\;+\;T\;-\;C\;\times \;n\;\times \;V}\;\times 100\%$$

where *C* is the concentration measured of free CsA, *n* is dilution multiple, *V* is the total volume, *T* is the quantity of reagent for CsA, and *W* is the total weight of NVs.

To detect the releasing kinetic, CLs and MMCLs in PBS (1 mL) were placed in a dialysis bag (MWCO = 3500) and submerged in a 300 mL dissolution medium (PBS containing 0.5% Tween 80). All samples were placed in a 37 °C orbital shaker and stirred at 100 rpm for 48 h. At each time point, 1 mL dissolution medium was removed for HPLC analysis and another 1 mL fresh medium was replenished.

### Long‐Time Stability Measurement

1 mg mL^−1^ CLs and MMCLs were suspended in 1× PBS, DMEM, and 10% FBS, respectively, and stored at low temperature (4 °C) or physiological temperature (37 °C) conditions. Their hydrodynamic size and PDI were measured and monitored for a prolonged time by DLS analysis.

### Evaluation of Membrane Fusion Between CLs and MSC Membranes

For DiO labeling of CLs, 20 µL of 100 µm DiO (Meilun, Dalian, China) was dissolved in phosphatidylcholine‐containing chloroform to form the thin film. For DiI labeling of MSC membranes, 1 µm DiI (Meilun, Dalian, China) was used before the co‐extrusion process. A total of 5 × 10^4^ AML12 cells were cultured in a 48‐well plate and treated with MMCLs labeled with DiO and DiI. Following incubation for 2 h, cells were washed with PBS and the colocalization between CLs and MSC membranes was observed under the CLSM (LSM‐780, Carl Zeiss, Germany). The Pearson's correlation coefficient was analyzed and measured with ImageJ software.

In order to quantitatively evaluate the fusion efficiency, 10% of DSPE‐PEG‐FITC (Ruixi, Xi'an, China) was dissolved in phosphatidylcholine contained chloroform to form FITC‐labeled CLs (CLs‐FITC). The free DSPE‐PEG‐FITC was separated by ultracentrifugation in a SW41 Ti rotor (Beckman Coulter, USA) at 100 000 × g for 1 h. After the co‐extrusion process, the fluorescence signal and particle count of fused hybrid MMCLs were detected by a Flow NanoAnalyzer model (NanoFCM Inc., Xiamen, China). According to the accurate count results of CLs, MSC membranes, and MMCLs, the efficiency of membrane fusion was evaluated.

### Protein Analysis

The whole protein analysis of liposomes, MMs, and MMLs was performed by SDS‐PAGE. Total proteins were extracted using RIPA lysates (Beyotime, China) containing 1% protease inhibitor cocktail (APExBIO, USA) and equivalent micrograms of proteins were mixed with 4× loading buffer (Invitrogen, USA). All samples were heated at 100 °C for 10 min and loaded onto 12% bis‐tris protein gels. Following electrophoresis at 100 V (Bio‐Rad, USA), staining was performed with Coomassie brilliant blue (Biosharp, China). For western blot analysis, the protein was transferred onto PVDF membrane (0.22 µm, Millipore) and treated with primary antibodies against CXCR4 (60042‐1‐Ig, Proteintech, USA), and VLA‐4 subunit alpha (19676‐1‐AP, Proteintech, USA), and HRP conjugated anti‐rabbit IgG secondary antibody (ab205718, abcam, USA). Protein bands were detected by a digital chemiluminescence system (Bio‐Rad, USA).

### Inducing the Inflammation of Vascular Endothelium In Vitro

HUVEC was treated with 100 ng mL^−1^ TNF‐α (MedChemExpress, USA) for 4 h to establish the in vitro model of inflamed vascular endothelium according to the previous report.^[^ ^48^ ^]^ The mRNA expression of ICAM‐1, VCAM‐1, IL‐1β, and IL‐10 were assessed by quantitative real‐time PCR (qRT‐PCR). Before and after the TNF‐α treatment, HUVEC was washed three times with PBS. Total RNA was extracted from the cells by Trizol reagent and transcribed into cDNA for qRT‐PCR amplification. Table S1 (Supporting Information) lists the primers used in this study, and the primers were synthesized by Sangon Biotech (Shanghai) Co., Ltd.

### Chemotactic Migration of MMCLs In Vitro

A Transwell assay was applied to evaluate the migration ability of CLs and MMCLs. In brief, HUVEC was seeded on the upper chamber of the transwell (0.4 µm, Corning, USA) at the cell density of 2 × 10^4^ cells per well and incubated overnight. Then, the serum‐free medium containing 100 ng mL^−1^ TNF‐α was substituted for the medium in the bottom chambers for 4 h of activation. Meanwhile, CLs and MMCLs were incubated with 1 µm of fluorescent lipophilic tracer PKH26 (Sigma–Aldrich, USA) with the provided buffer at room temperature prior to ultracentrifugation at 100 000 × g for 1 h. Fluorescence was quantified using the SpectraMax i3X multi‐mode microplate reader (excitation at 560 nm, emission at 595 nm, cutoff at 590 nm). Subsequently, HUVEC in the upper chamber were washed thrice with PBS and incubated with PKH26‐labeled CLs or MMCLs with the same fluorescent intensity. At time points of 2, 4, and 8 h, medium in the bottom chamber was collected for the analysis of fluorescence intensity. The ratio of the fluorescence intensity of the medium in the bottom chamber to the initial fluorescence intensity of the medium in the upper chamber after the addition of NVs was defined as the percentage of NVs that crossed the endodermis. To investigate the abilities of hepatic delivery of CLs and MMCLs after passing through the HUVEC monolayer, HUVEC (2 × 10^4^ cells per well) and AML12 cells (1 × 10^5^ cells per well) were seeded into the upper chamber and the bottom chamber of Transwell plate, respectively. PKH26‐labeled CLs or MMCLs were added into the upper chamber and incubated with HUVEC for 2 or 4 h. Then, AML12 cells were washed three times using PBS and stained with Hoechst 33342. The fluorescence signal of cells was detected through CLSM (LSM‐780, Carl Zeiss, Germany).

### Inhibition Experiment

To investigate the roles of CXCR4 and VLA‐4 in chemotactic migration, specific inhibitors AMD3100 (HY‐10046, MCE, USA) for CXCR4 and BIO5192 (HY‐107589, MCE, USA) for VLA‐4 were utilized. The experimental design included control groups, AMD3100‐treated group, BIO5192‐treated group, and a combination treatment group with both AMD3100 and BIO5192. An inhibition experiment was conducted using an in vitro model of transendothelial migration. PKH26‐labeled CLs and MMCLs were pre‐incubated with AMD3100 at 10 µm or BIO5192 at 2 µm at 37 °C for 30 min. Subsequently, the NVs/inhibitor mixtures were loaded in the upper chambers. 4 h later, the medium from the lower chamber was collected and analyzed for fluorescence intensity to assess migration.

### In Vitro Hepatocyte H/R Model

To induce in vitro ischemia injury, AML12 cells were incubated with HBSS in an atmosphere containing 95% N_2_ and 5% CO_2_ at 37 °C to mimic ischemia for 8 h. Reoxygenation was subsequently achieved by replacing HBSS with a complete medium and placing cells in an incubator containing 95% air and 5% CO_2_ at 37 °C for 6 h.

### Cell Viability Detection

AML12 cells were seeded in 96‐well plates at a density of 5 × 10^3^ per well. To evaluate the protective effect of free CsA, CLs, and MMCLs on the H/R injured cells, drug treatment was carried out at the beginning of reoxygenation. After incubation with a range of CsA concentrations (0.1, 1, 10, 25, 50, 100, and 500 ng mL^−1^) of free CsA, CLs, and MMCLs, cell viability of the injured cells was assessed by a CCK‐8 kit (BD Pharmingen, USA). Additionally, to clearly determine whether the MMs offer protection against H/R injury, a separate group designated as the MMs group was established. The quantity of MSC membrane proteins used in this group is equivalent to that used in the corresponding MMCLs group. The blank group was AML12 cells undergoing the same H/R manipulation and treated with PBS instead of drugs. The absorbance at 450 nm was measured using a microplate reader (SpectraMax i3X, Molecular Devices, USA). Cell viability was expressed as a percentage relative to control cells without H/R treatment.

### Measurement of Mitochondrial Integrity and Intracellular ROS Levels

The H/R injured AML12 cells were treated with PBS, free CsA, CLs, and MMCLs with CsA concentration of 0.1 ng mL^−1^ at the beginning of reoxygenation. The control group was normal AML12 cells without H/R manipulation and treated with PBS instead of drugs. JC‐1 fluorescence probe (Beyotime, China) was performed to detect the mitochondrial membrane potential according to the manufacturer's protocol. For intracellular ROS analysis, a DHE fluorescence probe (MedChemExpress, USA) was used according to the manufacturer's protocol, and the cell nucleus was stained by Hoechst 33342. All samples were washed with PBS and observed by CLSM (LSM‐780, Carl Zeiss, Germany). Also, the levels of superoxide generated by mitochondria were assessed using MitoSOX Red (ThermoFisher, USA), and the fluorescence signals in all samples were analyzed by the flow cytometry (CytoFLEXLX, Beckman Coulter, USA).

### Cell Apoptosis Detection

The H/R injured AML12 cells were treated with PBS, free CsA, CLs, MMs, and MMCLs with CsA concentration of 0.1 ng mL^−1^ at the beginning of reoxygenation.

The quantity of the membrane proteins in MMs group was comparable to that of the MMCLs group. The control group was normal AML12 cells without H/R manipulation and treated with PBS instead of drugs. To detect cell apoptosis after drug treatment, all cells were stained using the Calcein‐AM/PI double staining kit BestBio (Shanghai, China). Then all cells were analyzed through a high‐content analysis (HCA) system (PerkinElmer, USA). Furthermore, the annexin V‐FITC/PI dual staining kit (BD Pharmingen, USA) was used to detect the early and late apoptosis of the above samples according to the manufacturer's protocol. The fluorescence signals of cells were analyzed by flow cytometry (CytoFLEXLX, Beckman Coulter, USA). The excitation/emission wavelengths were 488 nm/525 nm for FITC and 535 nm/615 nm for PI.

### Animals

C57BL/6 mice (male, 6−8 weeks old, weighting ≈20 g) were purchased from Guangdong Yaokang Biotechnology Co., Ltd. (Guangdong, China). All mice were housed in the Experimental Animal Center of Ruiye Model Animal (Guangzhou) Biotechnology Co., Ltd (China) under specific pathogen‐free (SPF) conditions and were given care according to the Guideline of Sun Yat‐sen University for Animal Experimentation. Animals received free access standard laboratory diet and water, which were maintained in a constant environment, 50% humidity and 20 °C temperature, 12 h dark and light cycle.

### HIRI Mouse Model

All experimental processes of animals abided by the National Institutes of Health Guide for the Care and Use of Laboratory Animals and were approved by the Experimental Animal Ethics Committee of Ruiye Model Animal (Guangzhou) Biotechnology Co., Ltd, China, approval no. RYEth‐20230607256. The standard protocol of a mouse HIRI model was conducted as previously described.^[^ ^28^ ^]^ Briefly, mice were anesthetized with intraperitoneally 1% pentobarbital sodium at a dose of 30 mg kg^−1^. An atraumatic clip was used to interrupt the artery/portal vein blood supply to the left and middle liver lobes for an ischemic period of 90 min. Then, the atraumatic vascular clamp was removed for reperfusion. Sham‐operated mice underwent the identical procedure except for artery/portal vein occlusion.

### In Vivo Biodistribution of CLs and MMCLs

C57BL/6 mice conducted with HIRI operation were averagely dived into CLs and MMCLs groups (*n* = 5 per group). CLs and MMCLs were both labeled with 1 µm DiR fluorescence dye (Meilun, Dalian, China) and then injected into mice via the inferior vena cava immediately after the initiation of reperfusion. After different time intervals (2, 6, 24, and 48 h), in vivo, fluorescence signals were examined using the In Vivo Xtreme II small animal imaging system (Bruker, Germany). After that, the mice were euthanatized, and major organs including the heart, lung, liver, spleen, and kidney were separated for ex vivo imaging of the nanoparticles using the same imaging system. For immunofluorescence imaging, CLs and MMCLs were both labeled with 1 µm PKH26 (Sigma–Aldrich, USA) and then injected into mice via inferior vena cava immediately after the initiation of reperfusion. After 2 h, parts of the liver and spleen of the two groups were isolated from mice and embedded in an optimal cutting temperature compound (Servicebio, China) and stored at −80 °C. The frozen liver and spleen slices were obtained by a frozen slicer (Leica CM1950). After washing with PBS, sections were stained with DAPI for 15 min. Images of sections were acquired with CLSM (LSM 510, Carl Zeiss).

### Determination of CsA Using LC‐MS

Sample preparation: Blood (100 µL for each) and fresh tissues (50 mg for each) of major organs (lung, liver, and spleen) were collected from mice post‐euthanasia. After washing with ice‐cold PBS to remove blood and debris, tissues were homogenized in 1 mL of 0.05 m ZnSO_4_ extraction solution with methanol: water ratio of 1:1. The homogenate was chilled at −20 °C for 1 h followed by centrifuge at 14 000 rpm for 10 min at 4 °C. Collected 500 µL of the supernatant for concentration. The concentrated extract was re‐dissolved in 100 µL methanol and centrifuged at 14 000 rpm for 10 min at 4 °C to remove any particles. Standard Preparation: 1 mg CsA standard sample was accurately weighed into a 1 mL volumetric flask, dissolved in methanol, ensuring thorough mixing to achieve a 1 mg mL^−1^ stock solution, and stored at −80 °C. This stock was further diluted with methanol to create working solutions with varying concentrations. LC‐MS Analysis: The system used a Thermo Hypersil GOLD C18 column (2.1 × 100 × 1.9 µm, Thermo Scientific, USA). Mobile phase A was 0.1% formic acid in water and mobile phase B was a 1:1 mix of acetonitrile and isopropanol. The gradient started at 5% B, increased to 80% B over 4 min, held for 4 min, and then returned to 5% B within 2 min. The flow rate was 0.2 mL min^−1^, and the column temperature was 60 °C. Mass spectrometry was performed using an electrospray ionization (ESI) source in positive ion mode. The transitions monitored for CsA were m/z 1220 → 1202.839.

### Cytokines Measurement and Liver Function Evaluation

The blood of mice was collected into a glass container and allowed to clot at room temperature for 2 h. Serum was obtained by centrifugation at 150 × g for 5 min to sediment erythrocytes and then at 350 × g for 15 min. An automatic biochemical analyzer (7180‐ISE, Hitachi, Japan) was applied to examine the serum levels of AST, ALT, and LDH.

### H&E and Immunohistochemical Staining

Hepatic tissues were fixed in 4% paraformaldehyde, followed by dehydration in an ethanol gradient. After paraffin embedding, the samples were sectioned into 5 µm thickness slices and were processed for H&E staining. Histopathological evaluation was conducted using the Suzuki Score System^[^ ^49^ ^]^ and examined in a blinded fashion by an experienced pathologist (Table S2, Supporting Information). Immunostaining for F4/80 and Ly6G was carried out on paraffin sections using F4/80 antibody (Abcam, ab16911) and Ly6G antibody (Abcam, ab25377). Then they were developed using a biotinylated alkaline phosphatase‐conjugated secondary antibody and DAB substrate kits. Images of sections were acquired with an optical microscope (E100, Nikon, Japan). Immunohistochemical evaluation was conducted using ImageJ software to calculate the proportion of positive cells in total cells.

### TUNEL Staining

The apoptotic liver tissue cells were assessed using the DeadEnd Fluorometric TUNEL System (Promage, USA) according to the manufacturer's protocol. After washing with PBS, sections were stained with DAPI for 15 min. The apoptotic cells were visualized using a panoramic scanning microscope (TissudFAXS SL Spectra, TissueGnostics, Austria).

### Label‐Free LC‐MS/MS Experiment

In the label‐free experiment, MMs and liver tissue samples were lysed, and their proteins were extracted using a total protein extraction kit (KeyGENE, Jiangsu, China). Protein digestion was performed using the filter‐aided sample preparation (FASP) method as previously described.^[^ ^50^ ^]^ In simple terms, the protein supernatant was mixed with four volumes of ice‐cold acetone and incubated at −20 °C overnight. Precipitated proteins were pelleted by centrifugation at 16 000 × g for 10 min at 4 °C and then washed with 80% acetone. The proteins were suspended in 200 µL of UA buffer (8 m urea, 150 mm Tris‐HCl, pH 8.0; Sigma) and incubated at room temperature for 1 h. Next, 100 µL of 10 mm iodoacetamide (Sigma) was added and incubated in the dark for 30 min. The sample was washed twice with 200 µL UA buffer at 14 000 × g centrifugation for 10 min at room temperature. Then, 50 µL of trypsin working solution (5 µg of sequencing‐grade modified trypsin (1:50 w/w, Promega, USA) dissolved in 50 µL of ultrapure water) was added for digestion and incubated at 37 °C overnight. The digested samples were desalted on C18 Cartridges (EmporeTM SPE Cartridges C18, bed I.D. 7 mm, volume 3 mL; Sigma), concentrated by centrifugation at 14000 × g for 10 min, and reconstituted in 40 µL of 0.1% formic acid. For LC‐MS/MS analysis, an Easy‐NLC 1000 Liquid Chromatograph coupled to a Q Exactive mass spectrometer (ThermoFisher, USA) was used for a 120‐min run. Peptides were separated on an RP‐C18 analytical column at a flow rate of 300 nL min^−1^ over 120 min. After capillary separation, digested samples were analyzed using a Q Exactive mass spectrometer.

### Proteome Data Calculation and Analysis

The raw data were processed using Proteome Discoverer software (version 2.4.1.15, ThermoFisher, USA) and searched against the protein sequence database downloaded from UniProt (Swissprot). The Comet search parameters were set as follows: enzyme: trypsin, maximum missed cleavages: 2, instrument: ESI‐TRAP, precursor mass tolerance: ± 10 ppm, fragment mass tolerance: 0.05 Da, use average precursor mass: false, modification groups from Quan method: TMT 6 plex, dynamic modifications: oxidation; acetyl, static modifications, database pattern: decoy, peptide FDR: ≤ 0.01. The expressed proteins from each sample in each group were grouped, and the differentially expressed proteins were used for bioinformatics analysis. The quality control of the protein data and differential expression analysis were performed using the DEP package (version 1.22.0) in R software (version 4.3.1). Differentially expressed proteins were defined as those with a log2 fold‐change (logFC) greater than or equal to ± 0.26 (a 20% change in expression) and a *p*‐value less than 0.05. Gene annotation and enrichment analysis were conducted using Metascape (https://metascape.org/gp/index.html#/main/step1). Gene set enrichment analysis (GSEA) was conducted on the MM proteins using the clusterProfiler package (version 4.8.3). This analysis specifically employed Gene Ontology Biological Process (GO BP) gene sets to determine enriched biological processes.

### Statistical Analysis

GraphPad Prism was used for statistical analysis. All data were represented as mean ± standard deviation (SD). Two‐tailed t‐test analysis was used for a comparison between two groups, and the differences among multiple groups were analyzed by one‐way ANOVA following Tukey's multiple comparisons tests. *p* values < 0.05 were considered significant.

## Conflict of Interest

The authors declare no conflict of interest.

## Supporting information

Supporting Information

## Data Availability

The data that support the findings of this study are available from the corresponding author upon reasonable request.
