# Supplementary material for: Mesenchymal Stem Cell Membrane‐Camouflaged Liposomes for Biomimetic Delivery of Cyclosporine A for Hepatic Ischemia‐Reperfusion Injury Prevention
Source: Adv Sci (Weinh). 2024 Jun 20;11(32):2404171. doi: 10.1002/advs.202404171 (PMC11348201; doi:10.1002/advs.202404171)
Supplement: Supplementary file 1 — Supporting Information [file ADVS-11-2404171-s001.pdf]

ADVANCED SCIENCE
Open Access

Supporting Information

for *Adv. Sci.*, DOI 10.1002/advs.202404171

Mesenchymal Stem Cell Membrane-Camouflaged Liposomes for Biomimetic Delivery of Cyclosporine A for Hepatic Ischemia-Reperfusion Injury Prevention

Haitian Chen, Wen Yin, Kang Yao, Jinliang Liang, Jianye Cai, Xin Sui, Xuegang Zhao, Jiebin Zhang, Jiaqi Xiao, Rong Li, Qiuli Liu, Jia Yao, Guohua You, Yasong Liu, Chenhao Jiang, Xiaotong Qiu, Tingting Wang, Qiang You, Yingcai Zhang, Mo Yang, Jun Zheng*, Zong Dai* and Yang Yang**

Supporting Information

Mesenchymal Stem Cell Membrane-Camouflaged Liposomes for Biomimetic Delivery of Cyclosporine A for Hepatic Ischemia-Reperfusion Injury Prevention

Haitian Chen, Wen Yin, Kang Yao, Jinliang Liang, Jianye Cai, Xin Sui, Xuegang Zhao, Jiebin Zhang, Jiaqi Xiao, Rong Li, Qiuli Liu, Jia Yao, Guohua You, Yasong Liu, Chenhao Jiang, Xiaotong Qiu, Tingting Wang, Qiang You, Yingcai Zhang, Mo Yang, Jun Zheng,* Zong Dai,* and Yang Yang**

Table S1. Sequences of the primers

Name	Sequences (5' − 3')
ICAM1 F1	ATGCCCAGACATCTGTGTCC
ICAM1 R1	GGGGTCTCTATGCCCAACAA
VCAM1 F1	TTTGACAGGCTGGAGATAGACT
VCAM1 R1	TCAATGTGTAATTTAGCTCGGCA
IL-1β F1	ATGATGGCTTATTACAGTGGCAA
IL-1β R1	GTCGGAGATTCGTAGCTGGA
IL-10 F1	GACTTTAAGGGTTACCTGGTTG
IL-10 R1	TCACATGCGCCTTGATGTCTG
TNF-α F1	GAGGCCAAGCCCTGGTATG
TNF-α R1	CGGGCCGATTGATCTCAGC
IL-6 F1	GGTACATCCTCGACGGCATCT
IL-6 R1	GTGCCTCTTTGCTGCTTTCAC
IL-18 F1	AGTTCTCTTCGTTGACAA
IL-18 R1	GTCCTCTTACTTCACTGT
CCL12-F1	ATTTCCACACTTCTATGCCTCCT
CCL12-R1	ATCCAGTATGGTCCTGAAGATCA
CCL-25 F1	AATTATCACCAGCAGGAA
CCL25-R1	TCACATTCATGTCCTCTG

Table S2. Standard of Suzuki score for liver injury

Score	Congestion	Vacuole degeneration	Necrosis
0	None	None	None
1	Slight	Slight	Single cell
2	Mild	Mild	< 30%
3	Moderate	Moderate	31 – 60%
4	Severe	Severe	> 60%

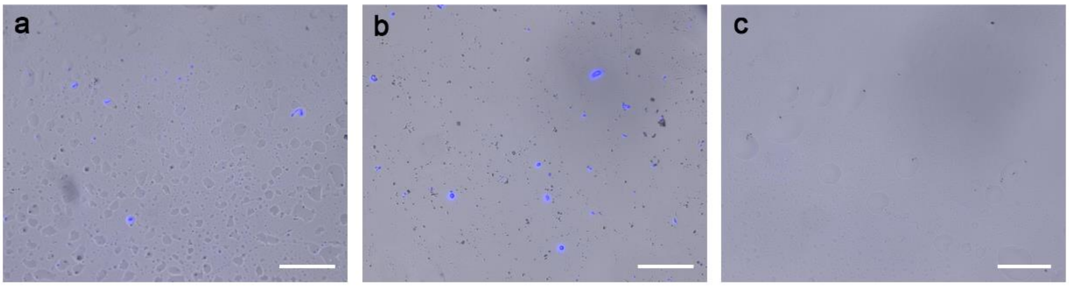

Figure S1. Inverted fluorescence microscopic images of (a) MSCs after freeze-thaw, (b) pellets of cell nuclei after centrifugation at 700 g for 10 min, and (c) pellets of MSC membranes after centrifugation at 15000 g for 30 min. Cell nuclei were pre-stained by DAPI. Scale bar: 100 μm.

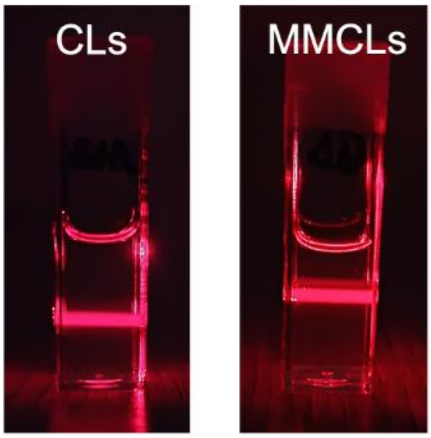

Figure S2. Tyndall effect of CLs and MMCLs after 30 days of storage at 4 ^{o}C in PBS.

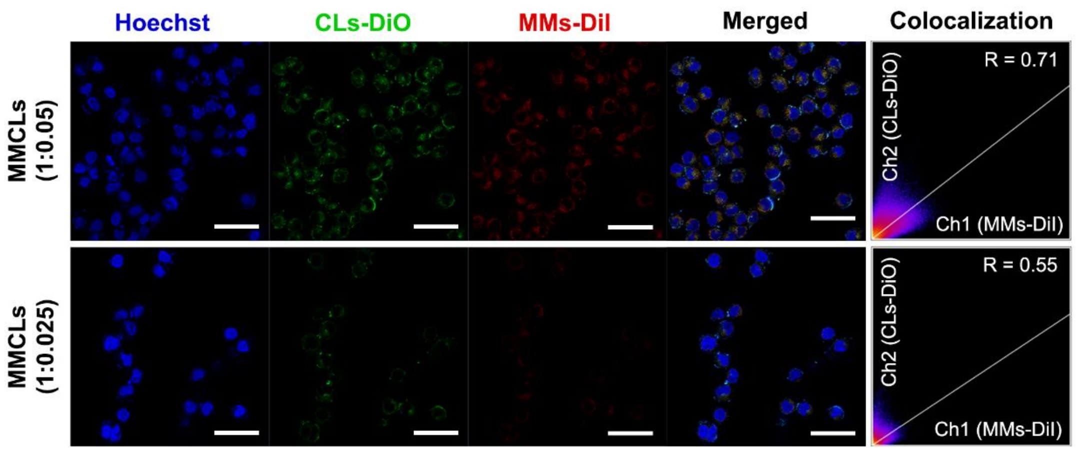

Figure S3. Colocalizations of CLs and MMs at phospholipid-to-membrane protein ratios of 1:0.05 and 1:0.025 after incubation with AML12 cells for 2 h and observed by CLSM. Scale bar: 50 μm.

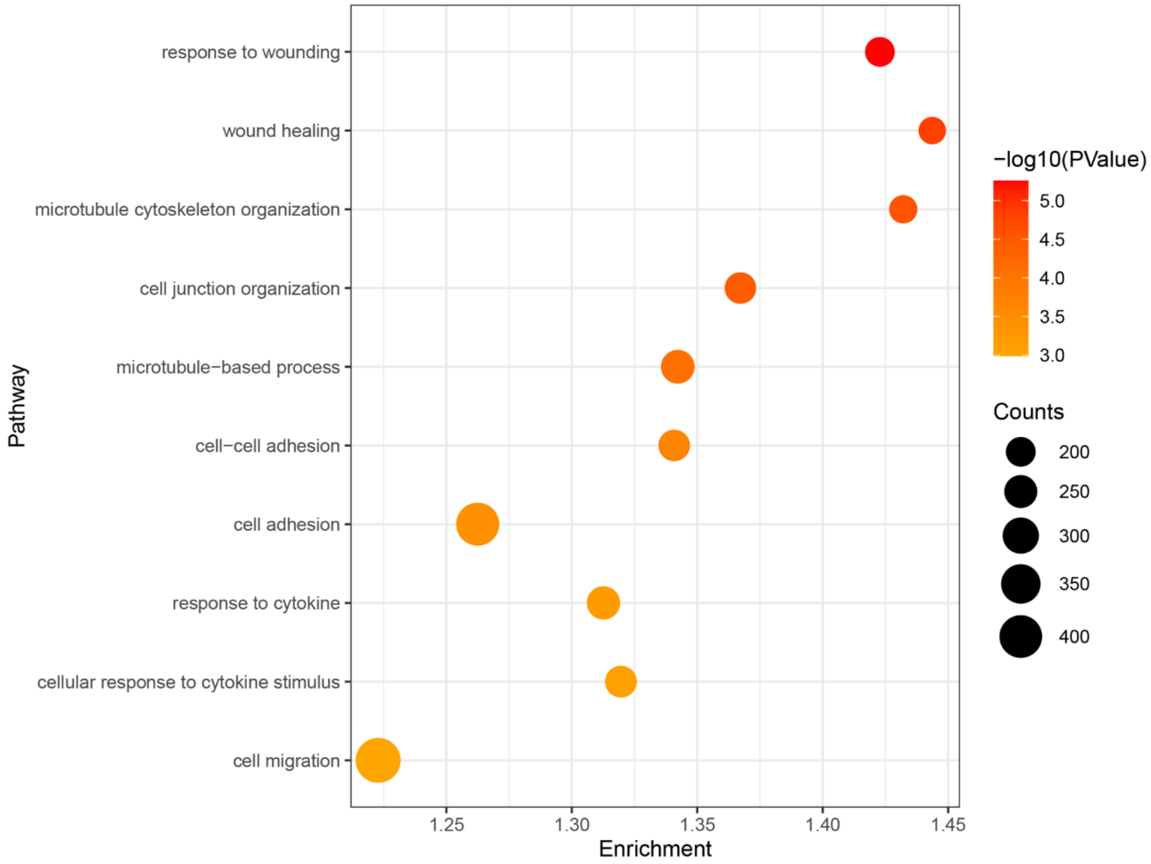

Figure S4. Functional enrichment analysis of the differently expressed proteins in MMs.

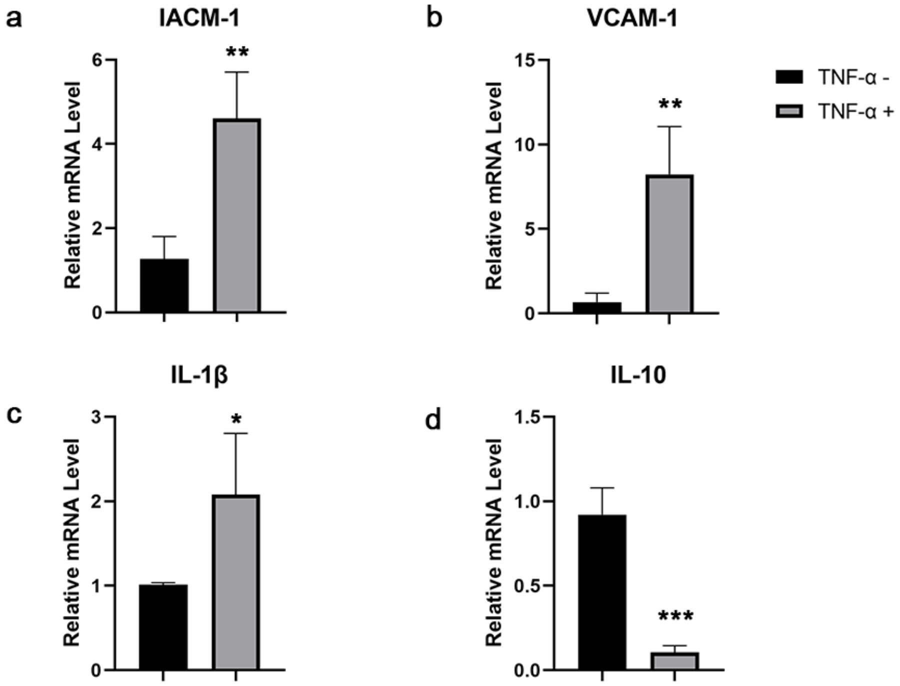

Figure S5. Relative mRNA expression of (a) ICAM-1, (b) VCAM-1, (c) IL-1β, and (d) IL-10 before and after TNF-α stimulation. Data are analyzed using two tailed t-test, *$p < 0.05$; **$p < 0.01$; ***$p < 0.001$ ($n = 4$).

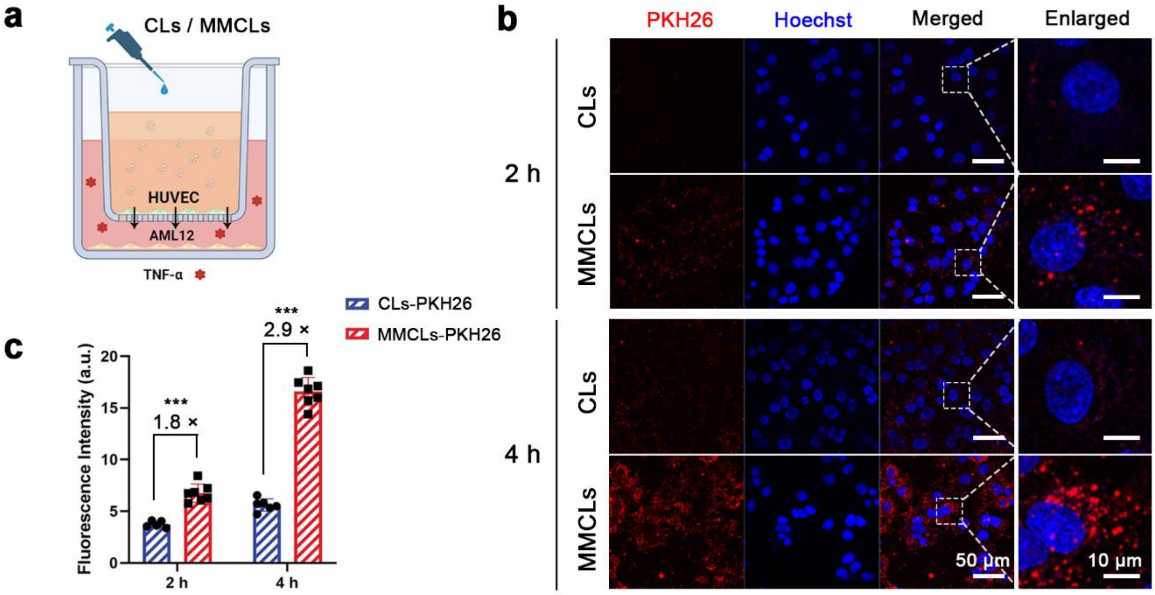

Figure S6. (a) Schematic diagram of transendothelial migration in Transwell. (b) CLSM images of AML12 cells in the lower chamber at 2 and 4 h after the addition of PKH26-labeled CLs or MMCLs with cell nuclei stained blue (Hoechst 33342). (c) The mean fluorescence intensity of

PKH26 delivered into AML12 cells. Data are analyzed using two tailed t-test, ***$p < 0.001$ ($n = 7$).

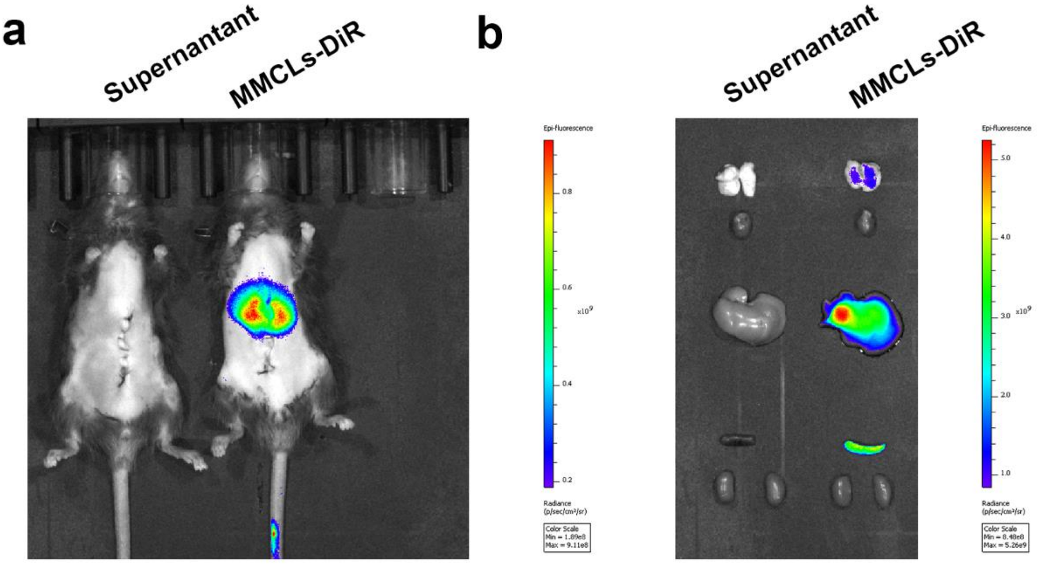

Figure S7. (a) In vivo fluorescence imaging of the supernatant and pellet of DiR-labeled MMCLs (MMCLs-DiR) in a HIRI mouse model at 6 h after injection. (b) Ex vivo tissue distribution of the supernatant of MMCLs-DiR in the main organs. Excitation wavelength: 750 nm; emission wavelength: 780 nm.

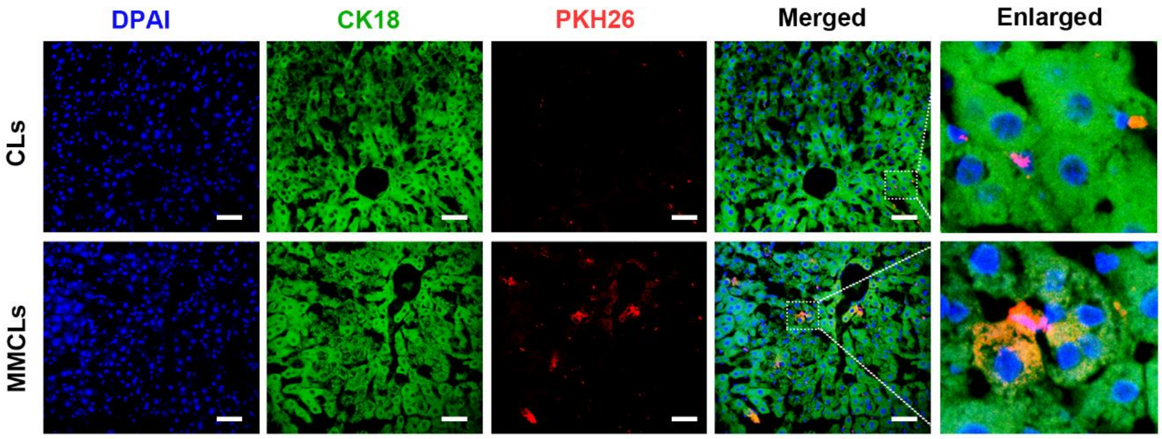

Figure S8. Immunofluorescence images of liver sections at 2 h after intravenous administration of PKH26-labeled CLs or MMCLs to HIRI mice, with cell nuclei stained blue (DAPI) and hepatocytes stained green (CK18). Scale bar: 50 μm.

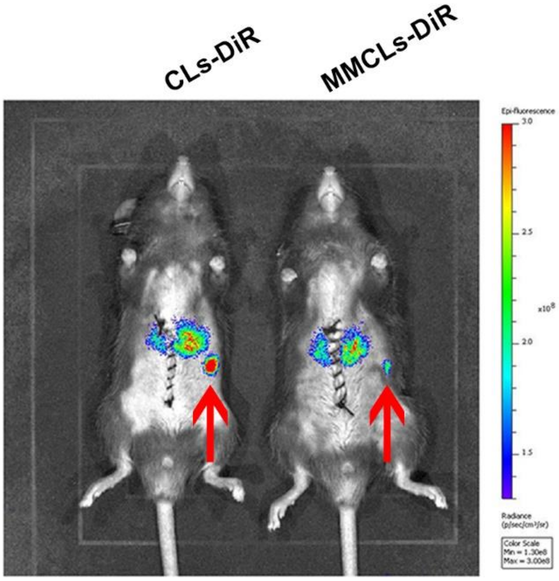

Figure S9. In vivo fluorescence imaging of DiR-labeled MMCLs or CLs in a HIRI mouse model at 2 h after reperfusion. Red arrows: spleen. Excitation wavelength: 750 nm; emission wavelength: 780 nm.

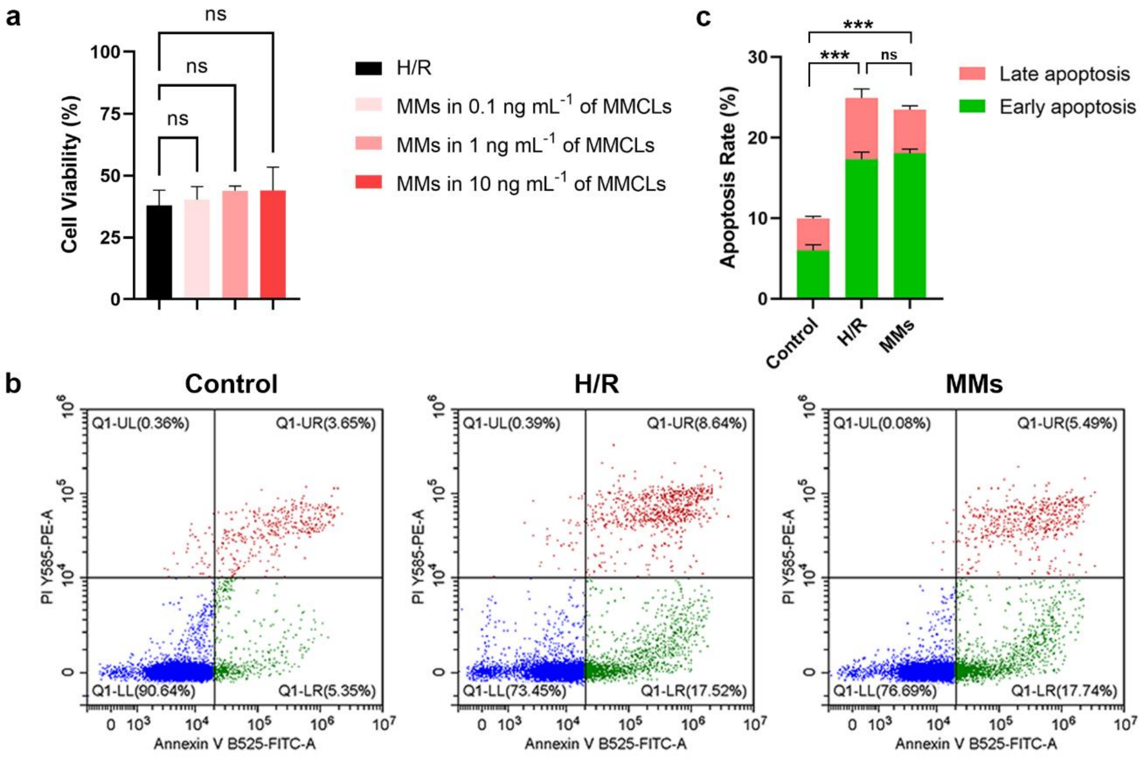

Figure S10. Evaluation of the protection effect of MMs on H/R-injured AML12 cells in vitro. (a) Cell viability of H/R-injured cells after treated with MMs equivalent to protein dose of MMCLs at CsA concentrations of 0.1, 1, and 10 ng mL^{-1} as measured by CCK-8 assay (n = 5). (b) Flow cytometry analysis of cell apoptosis of control, H/R-injured, and MMs-treated (equivalent to MMCLs at CsA concentrations of 0.1 ng mL^{-1}) groups by annexin V-FITC/PI

dual staining assay, and (c) the corresponding rates of early and late apoptosis ($n = 3$). Data are presented as mean ± SD. The statistical significance was analyzed using one-way ANOVA following Tukey's multiple comparisons test (*$p < 0.05$; **$p < 0.01$; ***$p < 0.001$, ns = no significance).

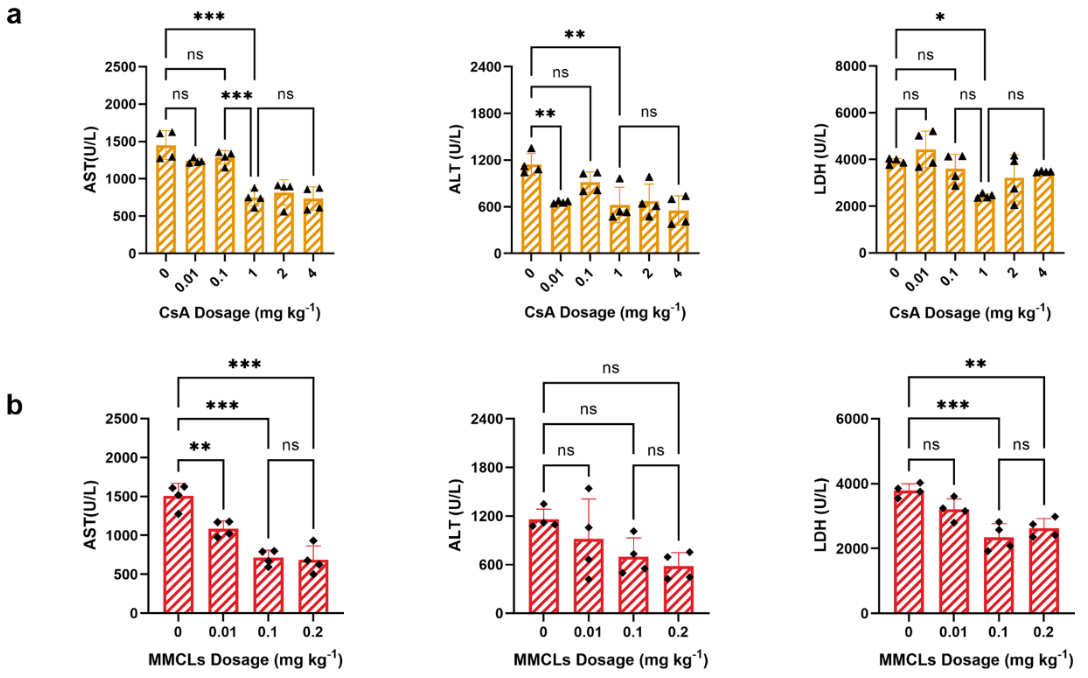

Figure S11. Evaluation of liver functions of AST, ALT, and LDH after administration of (a) CsA or (b) MMCLs at different CsA dosage in HIRI mouse model. Data are presented as mean ± SD. The statistical significance was analyzed using one-way ANOVA following Tukey's multiple comparisons test ($n = 4$, *$p < 0.05$; **$p < 0.01$; ***$p < 0.001$, ns = no significance).

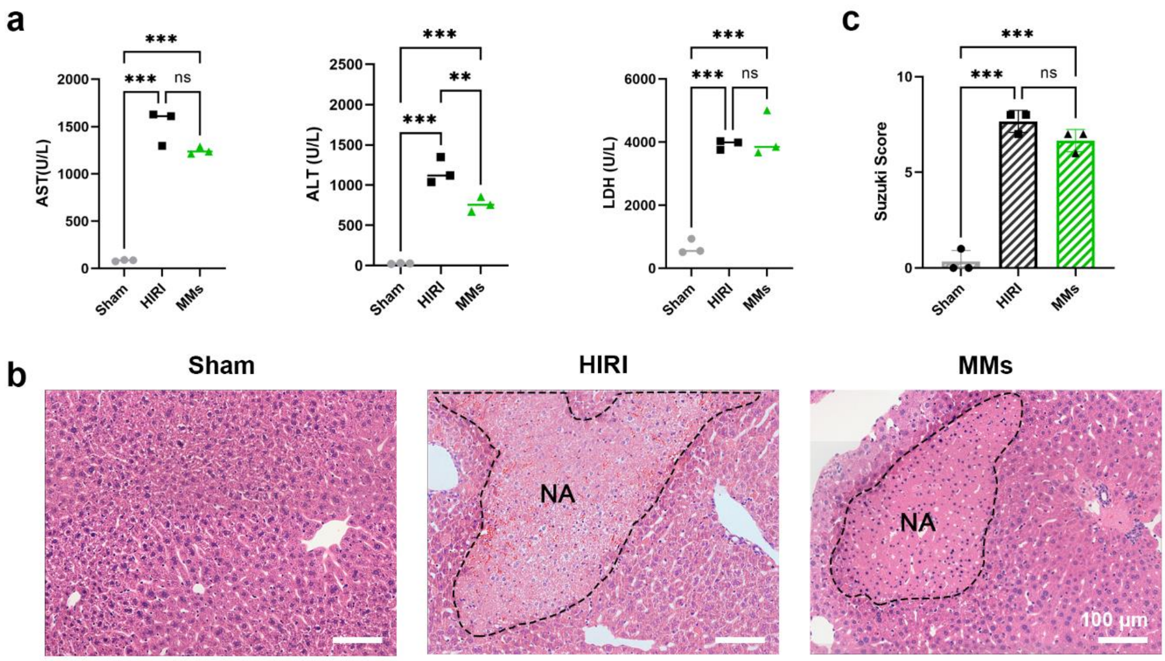

Figure S12. Evaluation of the protective effect of MMs. (a) Liver functions of AST, ALT, and LDH and (b) H&E staining of liver sections after administration with MMs at a protein dosage equivalent to 0.1mg kg^{-1} of MMCLs. (c) Suzuki scores of the H&E staining images. Data are presented as mean ± SD. The statistical significance was analyzed using one-way ANOVA following Tukey's multiple comparisons test (n = 3, *p < 0.05; **p < 0.01; ***p < 0.001, ns = no significance).

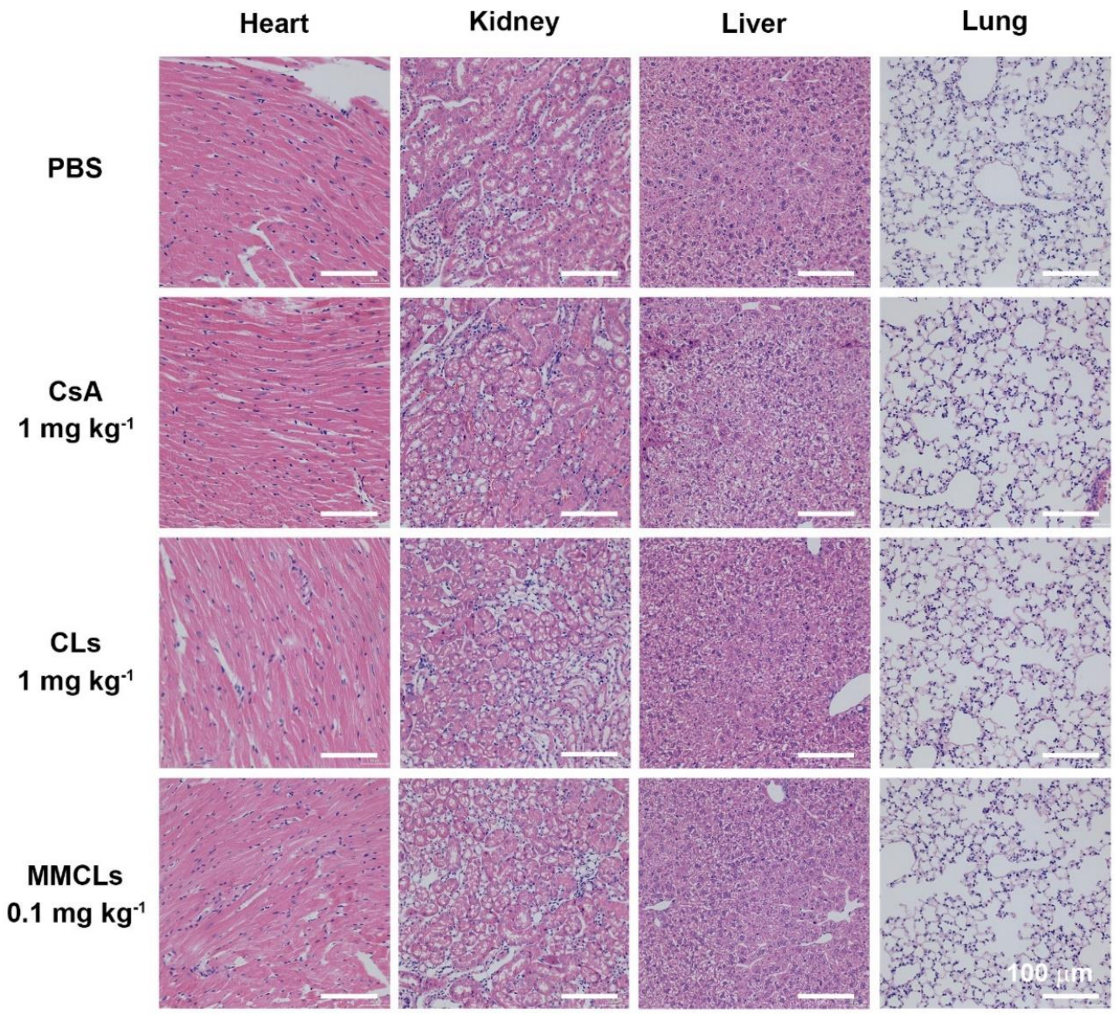

Figure S13. H&E staining of major organs including the liver, kidney, heart, and lung of healthy mice after treatment with PBS, CsA or CLs at CsA dose of 1 mg kg^{-1}, or MMCLs at CsA dose of 0.1 mg kg^{-1}.

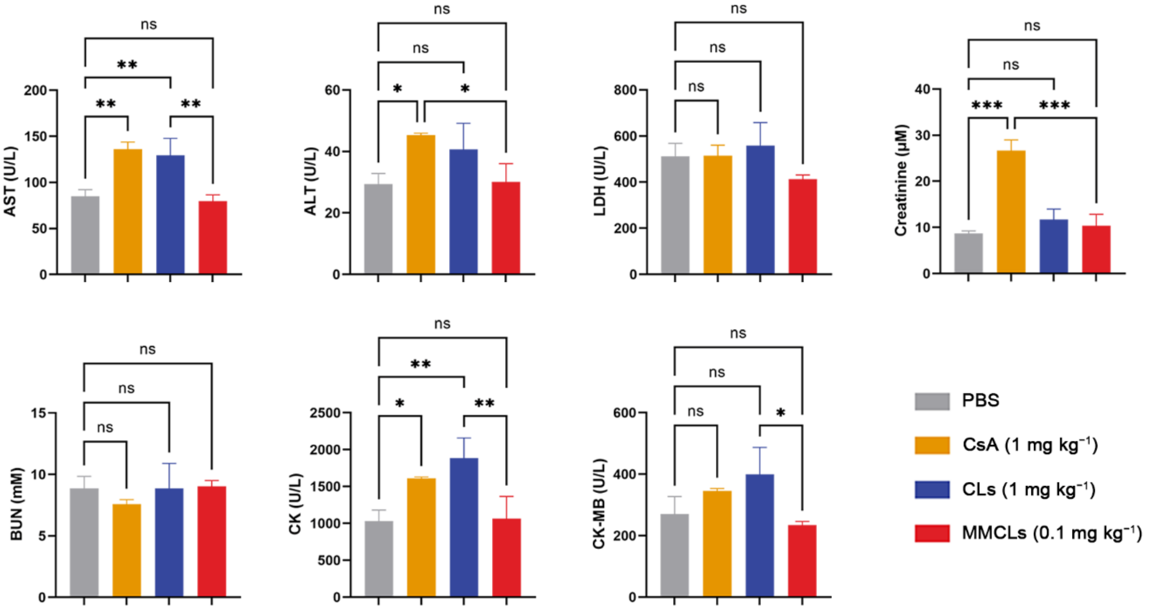

Figure S14. Detection of function indicators of liver (AST, ALT, and LDH), kidney (Creatinine and BUN), and heart (CK and CK-MB) in healthy mice. Data are presented as mean ± SD. The statistical significance was analyzed using one-way ANOVA following Tukey's multiple comparisons test (n = 3, *p < 0.05; **p < 0.01; ***p < 0.001, ns = no significance).

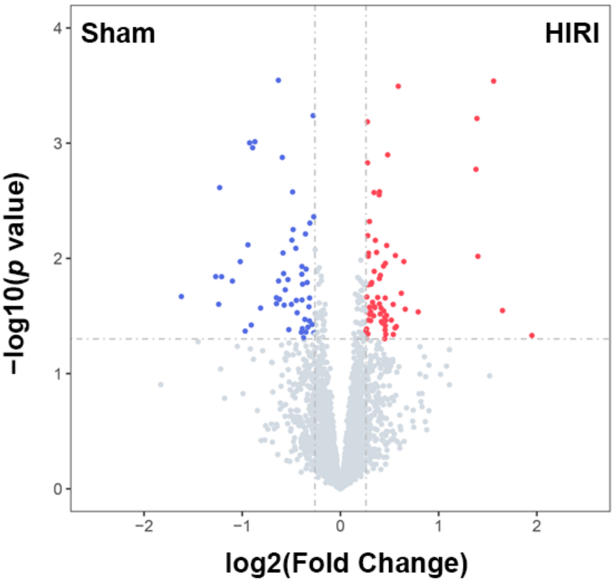

Figure S15. Volcano diagram showing the differently expressed proteins between the Sham group and the HIRI group, fold change > 1.2 or < 0.8, p < 0.05 (n = 5).

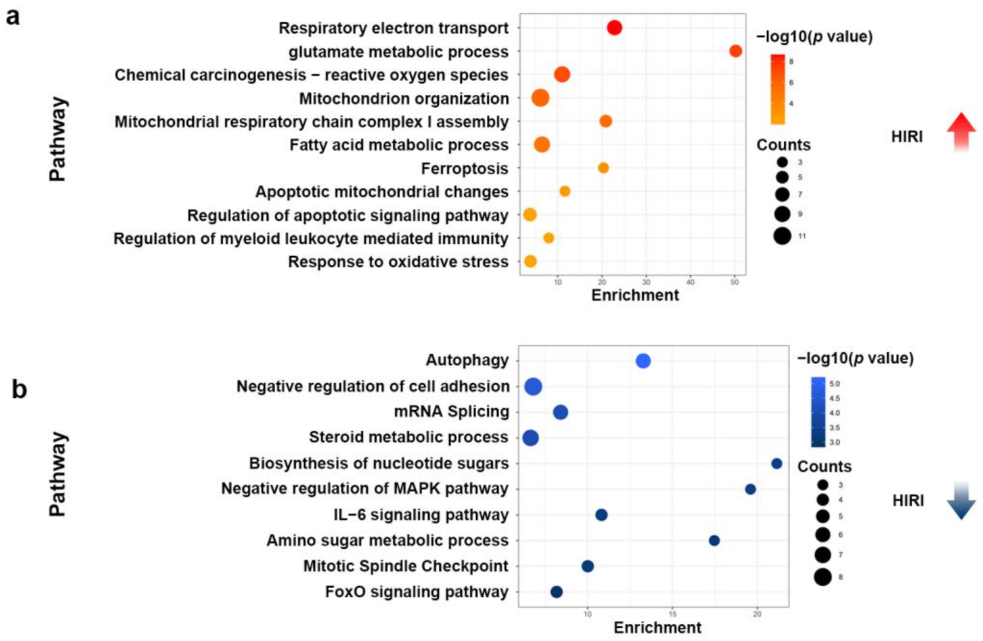

Figure S16. Functional enrichment analysis of the differently expressed proteins (a) upregulated or (b) downregulated in the HIRI group compared to the Sham group (*n* = 5).
